# Observation Bias in Metabarcoding

**DOI:** 10.1111/1755-0998.14119

**Published:** 2025-05-15

**Authors:** Megan R. Shaffer, Elizabeth Andruszkiewicz Allan, Amy M. Van Cise, Kim M. Parsons, Andrew Olaf Shelton, Ryan P. Kelly

**Affiliations:** ^1^ School of Marine and Environmental Affairs University of Washington Seattle Washington USA; ^2^ Conservation Biology Division Northwest Fisheries Science Center, National Marine Fisheries Service, National Oceanic and Atmospheric Administration Seattle Washington USA; ^3^ School of Aquatic and Fishery Sciences University of Washington Seattle Washington USA

**Keywords:** amplification efficiency, droplet digital PCR, environmental DNA, observation bias, quantitative metabarcoding

## Abstract

DNA metabarcoding is subject to observation bias associated with PCR and sequencing, which can result in observed read proportions differing from actual species proportions in the DNA extract. Here, we amplify and sequence a mock community of known composition containing marine fishes and cetaceans using four different primer sets and a variety of PCR conditions. We first compare metabarcoding observations to two different sets of expected species proportions based on total genomic DNA and on target mitochondrial template DNA. We find that calibrating observed read proportions based on template DNA concentration is most appropriate as it isolates PCR amplification bias; calibration with total genomic DNA results in bias that can be attributed to both PCR amplification bias and differing ratios of template to total genomic DNA. We then model the remaining amplification bias and find that approximately 60% can be explained by inherent species‐specific DNA characteristics. These include primer‐template mismatches, amplicon fragment length, and GC content, which vary somewhat across Taq polymerases. Finally, we investigate how different PCR protocols influence community composition regardless of expected proportions and find that changing protocols most strongly influence the amplification of templates with primer mismatches. Our findings suggest that using primer‐template pairs without mismatches and targeting a narrow taxonomic group can yield more repeatable and accurate estimates of species' true, underlying DNA template proportions. These findings identify key factors that should be considered when designing studies that aim to apply metabarcoding data quantitatively.

## 1 Introduction

With the rapid advancement of molecular methods in recent decades, it is now possible to easily characterise multiple species present in a single DNA sample. Leveraging conserved regions of the genome, specifically designed oligonucleotides, or primers, can amplify a diverse array of taxa, generating millions of sequences representing many different species. This approach, termed “DNA metabarcoding,” is often used to make inferences about the composition of the sampled community. Research on environmental DNA (eDNA) in particular has taken advantage of this technology (Taberlet et al. 2012), where there has been an exponential increase over the last decade of studies identifying DNA in soil, air, and/or water samples to characterise species compositions of terrestrial, marine, and freshwater environments (e.g., Miya 2022; Schenekar 2023; Shea et al. 2023).

**FIGURE 1:**
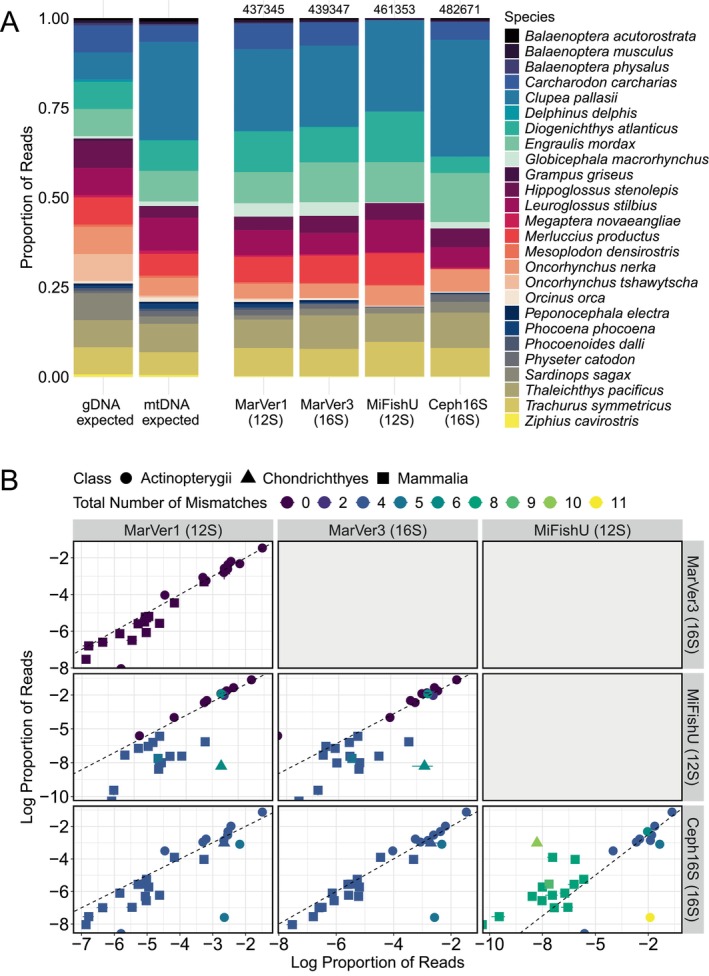
Metabarcoding output for the mock community, amplified using the Taq polymerase NEB Phusion HiFi. (A) The proportion of reads for each species in the mock community for each primer set (with three technical replicates averaged for each bar and total read depth for three replicates reported above bars; see Figure S4 for illustration of technical replication), compared to the expected proportions based on genomic DNA concentrations (via the Qubit Fluorometer) or mitochondrial DNA proportions (via ddPCR for MarVer1). (B) Comparison of log read proportions for all pairwise combinations of primer sets, with class and total numbers of mismatches (summed across both forward and reverse primers in the primer pair) between primers and template indicated by shapes and colours, respectively. The dotted line represents the 1:1 line.

eDNA metabarcoding is a powerful method for surveying species both quickly and relatively cheaply (Fonseca et al. 2023; Ruppert et al. 2019), and because of this, it is becoming a critical tool for monitoring global losses and shifts in biodiversity forecasted due to climate change (Deiner et al. 2021; Gallego et al. 2020; Lacoursière‐Roussel et al. 2018; Wilkinson et al. 2024). However, as with any other method of ecological monitoring, metabarcoding is prone to observation biases (Deiner et al. 2017; Krehenwinkel et al. 2017; Silverman et al. 2021; van der Loos and Nijland 2021). We observe metabarcoding data only at the end of a long chain of sampling and analytical processes, which each contribute to biases (e.g., see Figure 1 in: Gold et al. 2023; Harper et al. 2019; Shelton et al. 2016; Suarez‐Bregua et al. 2022). As a result, metabarcoding output in the form of sequence‐read proportions does not necessarily reflect the true proportions of species in the sampled environment (defined by species composition in units of biomass, counts, etc.). Furthermore, because metabarcoding datasets are inherently compositional (Gloor et al. 2017), and proportions of species must sum to one, when one species is underestimated in the data, others by definition must be overestimated. Therefore, the effects of amplification bias on a given species will inevitably affect the observations of all other species in the data (McLaren et al. 2019).

Among eDNA metabarcoding users, there consequently remains uncertainty as to whether or how proportions of sequence reads relate to proportions of species in a given sample or in a given environment. There has been considerable work relating metabarcoding reads to bacterial, fungal, and arthropod abundance and biomass (e.g., Edgar 2017; Jusino et al. 2019; Krehenwinkel et al. 2017; Krehenwinkel et al. 2018; McLaren et al. 2019; Palmer et al. 2018; Silverman et al. 2021; Sipos et al. 2007; Tedersoo et al. 2017; van der Loos and Nijland 2021 and more), and these methods typically involve capturing the whole organism from the environment for metabarcoding. Equivalent information for macroinvertebrate and vertebrate taxa—in which only traces of genetic material shed by the organism are collected—is thinner. Reports of strong relationships between read counts/proportions and fish biomass, abundance, and DNA concentrations suggest that there might be little to no observation bias in metabarcoding for some taxonomic groups or primer sets (e.g., Di Muri et al. 2020; Stoeckle et al. 2022); however, weak relationships between expectations and observations are also commonly reported for vertebrates and invertebrates (e.g., see Lamb et al. 2019). Dominant taxa can drive the strength of the relationship between reads and species, and changing species' abundances for only a few species in the composition can also alter this relationship (e.g., Skelton et al. 2022). Due to such uncertainty, many current studies have concluded that our limited understanding of metabarcoding requires further research before we can confidently use these data quantitatively, and that metabarcoding data should maybe only be used for presence/absence metrics (Elbrecht and Leese 2015). However, in order for eDNA metabarcoding results to be useful in a quantitative framework, we must identify the mechanistic processes that give rise to biased observations, understand when these occur, and develop both laboratory and statistical methods to account for them (e.g., Macher et al. 2023; van der Loos and Nijland 2021).

A common goal of eDNA metabarcoding users is to be able to translate sequence reads to quantitative information about multicellular species in the environment. However, the link between the abundance of organisms and the DNA present in the environment is complex. DNA can be shed at different rates depending on an organism's behaviour, life stage, size, and/or abundance (Andruszkiewicz Allan et al. 2021; Jo et al. 2019; Ostberg and Chase 2022; Thalinger et al. 2021; Wilder et al. 2023). Decisions around the collection of eDNA (e.g., pore size, filter type, volume filtered) and the subsequent metabarcoding workflow (e.g., DNA extraction, PCR, sequencing) can further influence the metabarcoding results (e.g., Andruszkiewicz Allan et al. 2021; Bessey et al. 2020; Deiner et al. 2018). Here, we focus on determining the causes of observation bias in the parts of the workflow from amplification of DNA to the acquisition of reads after sequencing, specifically focusing on the link between metabarcoding and eDNA concentration (not biomass of the organism from which it came), acknowledging that many factors control how eDNA relates to organismal abundance itself.

### 1.1 Measuring Observation Bias

Shelton et al. (2023) provide a means of measuring and correcting for amplification bias in metabarcoding, drawing on earlier work (McLaren et al. 2019; Silverman et al. 2021). In a single‐species context, the expected number of amplicons, A, is a function of the number of template copies present, c, amplification efficiency, a (defined as the fraction of target molecules copied from one cycle to the next; a value between 0 and 1), and the number of PCR cycles, $N_{\mathrm{PCR}}$ :

$$A\;=\;c{\left(1+a\right)}^{N_{\mathrm{PCR}}}$$

Given a known starting concentration and observed amplicon counts, it is therefore possible to estimate the amplification efficiency for a species‐primer pair—as is routinely done for quantitative PCR (qPCR).

In a multispecies context—that is, metabarcoding—different species amplify at different rates, and moreover, compete for limited reagents and sequencing‐read depth. Accordingly, we must evaluate the above parameters not as absolute values but as ratios, generally measuring changes for each of a set of species of interest relative to a common reference (see Shelton et al. 2023; McLaren et al. 2019; Silverman et al. 2021). We use one or more mock communities—that is, mixtures containing known DNA concentrations of extracts for known taxa—in combination with observed compositions of sequence‐reads after sequencing to estimate amplification efficiencies, $\alpha_{i}$, for each of the i species present (again, relative to an arbitrary reference species) and then to correct the estimates of the true (pre‐PCR) species proportions accordingly, as described in Shelton et al. (2023).

We note that $\alpha_{i}$ wraps up all species‐specific bias that occurs during PCR and sequencing and that we assume that the amplification efficiency is constant for a given species‐primer pair across community compositions (the baseline assumption of Shelton et al. 2023; McLaren et al. 2019; see Appendix S1: Section S5). Other examples in the literature of using mock communities to correct metabarcoding data share similar concepts and are equally helpful in understanding observation bias; see Edgar (2017), Palmer et al. (2018), Jusino et al. (2019), Silverman et al. (2021), and Moinard et al. (2023), among others.

Here, we distinguish and report sources of observation bias relating to both DNA concentration (total genomic DNA vs. template target DNA proportions) and amplification efficiency, which we discuss directly below.

#### 1.1.1 DNA Concentration

Accurately specifying the composition of a mock community—and therefore any resulting amplification biases—is contingent on correctly specifying DNA concentrations used for each species in the mock community. Importantly, there are at least two ways to quantify DNA concentration, which can yield different estimates of species compositions in the mock communities.

We can quantify DNA concentration either by total genomic DNA (gDNA; in ng/μL, usually measured by fluorometry) or by the number of copies of the target template (in copies/μL) in a sample. Template copy number can be measured by real‐time or qPCR, in which we compare an unknown sample to a standard curve of synthetic or amplicon DNA of known copy number (Conte et al. 2018; Taylor et al. 2010). However, comparing an unknown sample to the standard curve assumes that the assay is 100% efficient for the unknown sample. For species that do not amplify with 100% efficiency (as is often the case in eDNA metabarcoding), the given concentration from qPCR (i.e., Ct value) is conflated with the amplification efficiency. Digital or droplet digital PCR (d‐ or ddPCR), which divides the PCR reaction into thousands of partitions, can also yield quantitative estimates of template concentrations and is characterised as endpoint PCR, which relies less on amplification efficiency (Manoj 2014; Persson et al. 2019). Importantly, this method differs from qPCR insofar as it does not depend upon a standard curve, and thus estimates of template concentrations are more reliable and repeatable as they do not rely on standards that are prone to degradation nor on the construction of standard curves that are prone to human error and variability.

The total gDNA of an organism does not equal amplifiable template DNA because a given assay targets only a tiny fraction of the total DNA present. For example, primers targeting a locus of the mitochondrial DNA (mtDNA) genome should yield product as a function of the number of mtDNA genomes of the target taxa present in the sample extract, rather than as a function of the total mass of genomic DNA present. Thus, calibrating metabarcoding data using template copy number, rather than total genomic DNA, is the most relevant approach for studying bias due to the PCR process in a mock community context. In contrast, calibrating metabarcoding data using gDNA concentration results in estimated amplification efficiencies that contain biases due to both the ratio of template DNA to gDNA and the amplification efficiency; as a result, model calibration with gDNA concentrations will less accurately predict differences in PCR amplification efficiencies themselves.

#### 1.1.2 Amplification Efficiency

Because of the exponential PCR process, very small differences in amplification efficiency can yield enormous differences in proportional outcomes after 35–40 PCR cycles. Mismatches in the primer binding site between primer and template DNA can decrease the primer binding affinity and reduce efficiency, thereby causing bias against species that contain mismatches to the primer set (e.g., Piñol et al. 2014; Sipos et al. 2007; Wilcox et al. 2013). However, not all mismatches are created equal; for instance, mismatches near the 3′ of the primer are often more detrimental to amplification (Lefever et al. 2013). Similarly, the identity of the base in the mismatch also unequally affects amplification efficiency, likely driven by thermodynamics and polymerase performance (Rejali et al. 2018; Simsek and Adnan 2000; Stadhouders et al. 2010), as Taq polymerases have varying levels of fidelity.

Even when all taxa in a community contain perfect matches to the primer set, amplification bias can still exist due to other factors inherent to a species' DNA template. For instance, structural complexity and homopolymers or long stretches of repeat motifs occurring in or around the target often cause difficulty during primer binding, Taq polymerase elongation, and sequencing (Hansen et al. 1998; Peng et al. 2018; Shinde et al. 2003; Kieleczawa 2006).

Regions that contain high or low GC content (or the percentage of nucleotide bases in a DNA or RNA strand that are either quanine or cytosine) can lead to a reduced amplification efficiency during PCR (Benjamini and Speed 2012). Different Taq polymerases have been reported to show preference for specific GC content, and changing PCR protocols and cycling conditions has been shown to mitigate such bias (Laursen et al. 2017; Pan et al. 2014; Nichols et al. 2018). GC‐bias can also vary across different sequencing platforms, and coverage for most sequencing platforms greatly decreases outside optimal GC content ranges (Browne et al. 2020).

The length of the amplicon being amplified and sequenced can also cause bias in metabarcoding datasets. Shorter fragments amplify during PCR more efficiently than longer ones, and further, Taq polymerases can vary in performance to introduce length bias (Dabney and Meyer 2012). Length bias has also been reported during sequencing; for instance, smaller fragments preferentially bind to some types of sequencing flow cells. Sequencing platforms also vary in read length capabilities, with associated error rates varying across different read lengths as well (Murray et al. 2015). Typically, amplicon libraries are prepared for eDNA studies, and it is assumed that amplicons all contain approximately the same length. However, insertions or deletions may exist for a gene region across different taxonomic groups. Markers that capture multiple groups of taxa—which are often employed in eDNA studies—may therefore result in an amplicon library containing different size fragments (this length heterogeneity also has implications for bioinformatic processing [see Palmer et al. 2018]).

### 1.2 Study Objective

Here, we sequence a mock community containing fishes and cetaceans under various and replicated scenarios to determine the mechanisms driving observation bias in metabarcoding. We first compare calibration of the metabarcoding data with two methods of DNA quantification of mock community members (template vs. total genomic DNA), for four different primer sets and four different Taq polymerases. Leveraging the fact that the mock community members contained different primer‐template mismatches, GC content and fragment lengths, we then examine how such species‐specific DNA characteristics and Taq polymerase performances may contribute to amplification efficiency across the four primer sets. We finally examine how technical aspects of PCR may influence the community composition (regardless of expected proportions) by comparing different PCR protocols within one primer set. In doing this, we are able to identify some (but not all) key drivers of observation bias in metabarcoding datasets, which is an important step towards more quantitative, reliable, and interpretable results.

## 2 Materials and Methods

### 2.1 Experimental Design

#### 2.1.1 Mock Community Construction

We constructed the mock community using tissue extracts from 36 species, but only focus on 26 species for the subsequent analyses (see Appendix S1: Section S1.1 for full species list, DNA extraction information, entire mock community composition and reasoning for excluding some species; Tables S1 and S2, Figure S1). The mock community subset we use hereafter included fishes (including species in the Classes Actinopterygii and Chondrichthyes; *N* = 12) and cetaceans (Class Mammalia; *N* = 14) distributed throughout the California Current (Tables S1 and S2). All DNA extracts were sequenced following a standard Sanger sequencing protocol (Sanger 1981) to validate species' identity (see Appendix S1: Section S1.2 for primer sets used and Table S3 for GenBank Accession numbers for sequences deposited from this study). We quantified the genomic DNA of all extracts in triplicate with Qubit Fluorometer (Invitrogen) using the dsDNA high Sensitivity Assay Kit.

We then constructed two mock communities (with the following percentages recalculated for the mock community subset): (1) one that consisted of approximately 91% fishes and 9% cetaceans, with equal gDNA concentration of each species within each group (which we call the “even” mock community); and (2) one that consisted of approximately 91% fish and 9% cetaceans, but with fish and cetacean species at different gDNA concentrations within each group (which we call the “skewed” mock community). From hereafter, the mock community we refer to is the even mock community, but more information about the construction of, amplification of, and analyses performed on the skewed mock community can be found in Appendix S1. The concentration of final mock communities (3 ng/μL) was left intentionally high so that failed amplification could be attributed to differences in reaction conditions (e.g., primer bias, Taq performance, effect of PCR additives, etc.) rather than stochasticity of amplifying samples with low template concentration (e.g., Gold et al. 2023).

#### 2.1.2 Amplification of the Mock Community

We amplified the mock community along with a no template control (NTC) with four primer sets found in Table 1, which each target different taxonomic groups, including fish (MiFishU, MarVer1, MarVer3), cephalopods (Ceph16S), and marine mammals and vertebrates (MarVer1, MarVer3). We chose these primers because they are commonly used in eDNA metabarcoding studies, they target different genes (12S, 16S), and they vary in the scope of their targets (e.g., either a narrow group of taxa [e.g., MiFishU, Ceph16S] or broader groups of taxa [e.g., MarVer1, MarVer3]).

**TABLE 1:** Sequence information and references for all primers used in this study.

Primer	Targeted gene	Sequence	Ref
Ceph_16S_F	16S	GACGAGAAGACCCTAWTGAGCT	(1)
Ceph_16S_R	16S	AAATTACGCTGTTATCCCT	(1)
MiFish‐U‐F	12S	GCCGGTAAAACTCGTGCCAGC	(2)
MiFish‐U‐R	12S	CATAGTGGGGTATCTAATCCCAGTTTG	(2)
MarVer1F	12S	CGTGCCAGCCACCGCG	(3)
MarVer1R	12S	GGGTATCTAATCCYAGTTTG	(3)
MarVer3F	16S	AGACGAGAAGACCCTRTG	(3)
MarVer3R	16S	GGATTGCGCTGTTATCCC	(3)

*Note:* (1) Deagle et al. (2009); (2) Miya et al. (2015), note the forward primer contains a C as the second base pair; (3) Valsecchi et al. (2020).

We prepared amplicon libraries of the mock community using a two‐step PCR protocol. First, we amplified the mock community using primers with Illumina adapter overhang sequences (P5 overhang: 5′‐TCGTCGGCAGCGTCAGATGTGTATAAGAGACAG‐forward‐primer‐sequence‐3′; P7 overhang: 5′‐GTCTCGTGGGCTCGGAGATGTGTATAAGAGACAG‐reverse‐primer‐sequence‐3′). We amplified the mock community for the four different primer sets, for four different Taq polymerases ([1] Invitrogen Platinum Superfi [IPSF]; [2] NEB Phusion HiFi Taq [NPHF], [3] Promega GoTaq Flexi [PGTF]; [4] Qiagen Multiplex Master Mix [QMMM]), using the recipe and cycling conditions summarised in Tables S4 and S5. We performed a second indexing PCR where we uniquely indexed the product for each sample using unique dual indexes. We visually assessed the NTCs for each marker on a 2% agarose gel and included one NTC on the sequencing run to check that the absence of a visual band resulted in no reads. We sequenced our libraries on the Illumina MiSeq System located at the University of Washington. We loaded the final libraries at 8 pM with a 10% PhiX spike‐in and performed pair‐end sequencing using MiSeq Reagent Kits v3 (2 × 300). More detailed information for library preparation and sequencing can be found in Appendix S1: Section S2.1.

We then focused on one marker (MiFishU) to assess the effect of cycling conditions and PCR additives within a marker. We performed touchdown PCRs (TD‐PCRs) on the mock community for MiFishU with all four Taqs, following the cycling conditions in Min et al. (2021). We then assessed the effect of adding bovine serum albumin (BSA) by running additional PCRs for all Taqs, so that we had all MiFishU‐Taq combinations with and without BSA.

For each treatment, we ran triplicate reactions of the mock community and an NTC. In total, we performed library preparation and sequencing (as described above) for 24 treatments (see Table S6 for sequencing statistics and Figure S3 for a schematic of the treatments).

#### 2.1.3 Bioinformatic Analysis

We carried out all bioinformatic analyses in R (v 4.3.2; R Core Team 2022). We removed primers from sequences using cutadapt v1.18 (Martin 2011) and generated ASVs in the R package *dada2* (Callahan et al. 2016), which trimmed, filtered, and merged paired‐end reads. We determined truncation lengths for each of the amplified fragments (i.e., for all four primer sets) by visually assessing the aggregated quality score plots, and when merging, we used the default minimum overlap of 12 bp. The code for DADA2, which includes the settings we used, can be found at https://zenodo.org/records/12806658.

After ASVs were generated, we assigned taxonomy by comparing the ASVs to a downloaded version of NCBI's BLAST nucleotide nt database (downloaded 24 January 2024), using the blastn task (Altschul et al. 1990). We assigned species to the highest percent identity with a cutoff of 97%. While most ASVs were assigned algorithmically to a species in the mock, a small percentage of ASVs were curated based on knowledge of the mock community composition or based on the Sanger sequences generated for the mock extracts (see Section 2.1.4 in Apendix S1). For instance, if an ASV matched multiple species equally well, it was assigned to the species that was put into the mock community. Most species had one ASV per primer set; for those that had multiple, in most cases, the other ASVs were less than 1% of the total reads assigned to that species. None of the minor ambiguities in assigning taxonomy to ASVs from the mock community would substantially affect the results we report below.

### 2.2 Concentration of Total Versus Template DNA

#### 2.2.1 Template Concentration of Mock Species

To understand how template concentrations for mtDNA differed from gDNA in our mock community species, we performed ddPCR using an EvaGreen (Bio‐Rad) assay for four of our eDNA primer sets (MarVer1, MarVer3, MiFishU, Ceph16S; Table 1) for each individual species extract. We normalised each extract to approximately 0.05 ng/μL so that we could obtain estimates of copies/ng for each extract (see Appendix S1: Section S3). For each fish extract, we ran all four primer sets, and for cetaceans, we only ran MarVer1 and MiFishU (except for *Phocoena phocoena* , which we only amplified with MarVer1 due to extract limitation). We then used the copies/ng of each species's ddPCR extract to calculate the copies of each species added to our mock community (which we constructed based on ng).

We generated droplets using the Bio‐Rad QX200 AutoDG Droplet Digital PCR System, performed amplification in a deep‐well thermocycler, and read fluorescent droplets using the QX200 Droplet Reader. ddPCR recipe and cycling conditions can be found in Appendix S1: Section S3. We determined positive droplets by comparing the samples to two negative controls of DNA/RNAse free water to establish a baseline threshold of fluorescence, then considered droplets above negative baseline amplitude to be positive. We recalculated the proportion of species in the mock community using concentrations derived from ddPCR with MarVer1, treating these estimates as accurate estimates of mtDNA concentration (as this primer set contained no mismatches to any species in the mock community and routinely yielded estimates consistent with other primers without mismatches—see Section 3).

#### 2.2.2 Calibration with Quantitative Metabarcoding Model

To understand how observed amplification efficiencies differed between calibration methods, we calibrated our mock community proportions based on expected proportions from: (1) total gDNA concentration via Qubit Fluorometry and (2) template mtDNA concentration via ddPCR. We ran the quantitative metabarcoding model described by Shelton et al. (2023) for each marker using both gDNA and mtDNA as expected proportions. This model was implemented using the package “rstan” (Stan Development Team 2024), with Hamiltonian Markov Chain Monte Carlo sampling run using three chains, 500 warm‐up iterations and a total of 1500 iterations per chain. The model efficiently converged, with $\hat{R}$ values < 1.01 for all parameters.

### 2.3 Distinguishing Causes of Amplification Bias

To approximate different primer binding affinities for all species‐primer matches, we enumerated the mismatches between the forward and reverse primer binding sites for all fish and cetacean species and each of the four primer sets (MarVer1, MarVer3, MiFishU, Ceph16S). For each species, we downloaded all available reference sequences for each gene of interest (12S, 16S) and performed a virtual PCR with each associated primer set using the *insect* package (Wilkinson et al. 2018) in R. The virtual PCR returned trimmed sequences for each gene fragment with the primer binding site, which we aligned to determine the number of mismatches in Geneious Prime v 2024.0.3 (see Appendix S1: Section S4.1 for more information). For each species, we also determined the GC content and length of its amplicon (see Appendix S1: Section S4.2, Table S9), where GC content was calculated as the percent of nucleotides that were either a guanine or cytosine in the amplicon and fragment length was the length of the fragment in base pairs (both without the primer binding site).

#### 2.3.1 Modelling Amplification Efficiency due to DNA Characteristics

After determining that mtDNA template copy number performed better in calibrating species proportions (see Section 3), we then investigated the remaining bias by examining the effects of number of mismatches, GC content and amplicon length on species‐specific amplification efficiencies (derived from mtDNA calibration). To do this, we employed a linear regression model implemented in a Bayesian framework, using the “stan_lm” function from the “rstanarm” package (Goodrich et al. 2024) in R. To fit these models, we combined the species‐specific amplification efficiencies from all four markers (MarVer1, MarVer3, MiFishU, Ceph16S) with all Taqs polymerases (IPSF, NPHF, PGTF, QMMM). Because the amplification efficiency parameters are on a common scale within each marker and Taq polymerases—the estimated parameter is the log‐ratio of each individual species relative to that of an arbitrary reference species (here, *Engraulis mordax* )—it is possible to combine information across loci to create a generalisable analysis. We modelled amplification efficiency as follows:

(1) $$\alpha_{\mathrm{ij}k}=\beta_{1k}{\mathrm{mm}}_{\mathrm{ij}}+\beta_{2k}{\mathrm{GC}}_{\mathrm{ij}}+\beta_{3}{\mathrm{len}}_{\mathrm{ij}}+\varepsilon_{\mathrm{ij}k}$$

where $\alpha_{\mathrm{ij}k}$ is the overall amplification efficiency for species i at locus j for Taq k (again, expressed as the log‐ratio of efficiency for species i relative to the reference species, as described in Shelton et al. 2023), which is the quantity we wish to explain. $\beta_{1k}$, $\beta_{2k}$, and $\beta_{3}$ are coefficients for predictor variables; and $\varepsilon_{ijk}$ represents the error term $\varepsilon_{ijk}\sim N\left(0,\sigma_{ijk}\right)$. The predictor variables are as follows: ${\mathrm{mm}}_{ij}$ is the total number of mismatches summed across the forward and reverse primer binding sites; ${\mathrm{GC}}_{ij}$ is the GC content of the amplicon (without the primer binding site); and ${\mathrm{len}}_{ij}$ is the amplicon length (without the primer binding site) in base pairs. Note that ${\mathrm{mm}}_{ij}$, ${\mathrm{GC}}_{ij}$, and ${\mathrm{len}}_{ij}$ are not absolute values for each species, but rather expressed relative to the reference species *Engraulis mordax* , due to the compositional nature of metabarcoding data. For example, if species i had a total of three mismatches to the primer set at locus j and the reference species had one mismatch, ${\mathrm{mm}}_{\mathrm{ij}}$ would be expressed as 3–1 = 2. Information about model testing and selection can be found in Appendix S1: Section S5.1.

#### 2.3.2 Measuring the Effects of PCR Protocols on Community Composition

To investigate how different PCR protocols can affect amplification bias, we quantified the effects of Taq polymerase (IPSF, NPHF, PGTF, QMMM); PCR additives (BSA vs. no BSA); and cycling conditions (normal vs. TD cycling) on community composition using *zoid* (Jensen et al. 2022), an R package that implements a modified Dirichlet regression to handle compositional data with observations including 0 and 1. To examine the effects of different treatments within a single primer set, we first fit a linear model:

(2) $$Z_{ijkl}=\gamma_{0i}+\gamma_{1ij}+\gamma_{2ik}+\gamma_{3il}$$

where $Z_{ijkl}$ is the observed proportion or read count for species i, using Taq j, BSA treatment k, and cycling condition l; $\gamma_{0_{i}}$ is the species‐specific residual (intercept) term; and ${\gamma_{1}}_{ij}$, ${\gamma_{2}}_{ik}$ and $\gamma_{3_{il}}$ are the terms for each predictor. We fit this model on a subset of the data that contained reads generated with MiFishU for only Actinopterygii species (because the other species in the mock community were less than 0.55% of the MiFishU reads). As with Bayesian techniques in general, *zoid* does not reflect the test of a null hypothesis and therefore does not report *p*‐values. Instead, posterior credibility intervals reflect the plausibility of different parameter values; where these intervals indicate a parameter value is likely to be far from zero, that parameter is influential in the model. Note here we focus on whole community composition regardless of expected proportions, in order to highlight that sample in which expected proportions may not be known (like eDNA samples) may vary depending on PCR protocol.

## 3 Results

### 3.1 Metabarcoding the Mock Community

In total, we generated 16,144,143 reads across all samples (with each sample run in triplicate, see Figure S3; bioinformatic output and read depths for all samples and for all treatments can be found in Tables S7 and S8, respectively). For the four primer sets, 99.7% or more of the reads assigned to a species included in the larger mock community. Mean read proportions for the mock community subset for all four primer sets amplified using NEB Phusion HiFi Taq can be found in Figure 1A, and using all other Taqs can be found in Appendix S1: Section S2.2 (along with results of technical replication, see Figure S4). Species had comparatively little to no reads to marine mammal species when amplified with MiFishU versus MarVer1, MarVer3, and Ceph16. There were a very small number of ASVs that were not assigned to species in the mock, and more information on these off targets can be found in Appendix S1: Section S2.2.3.

Species' read‐proportions were very strongly correlated across primer sets where mismatches were absent (MarVer1 vs. MarVer3, Kendall's Tau, τ = 0.89, *p* = 3.331E‐15, *n* = 26; Figure 1B). Species' read‐proportions were invariably lower when amplified using primers with one or more mismatches when compared to primers without mismatches (e.g., MarVer1 vs. MiFishU, τ = 0.61, *p* = 3.866E‐06, *n* = 26; and MarVer3 vs. MiFishU, τ = 0.56, *p* = 2.896E‐05, *n* = 26; Figure 1C,E). In these cases, because the data are compositional, the species containing perfect matches were slightly overestimated. When the mock community members shared the same mismatch identity and position across a primer set (e.g., the case for Ceph16S), read composition resembled those of primer sets with perfect matches (e.g., MarVer1 vs. Ceph16S, τ = 0.67, *p* = 1.442E‐07, *n* = 26; and, MarVer3 vs. Ceph16S, τ = 0.71, *p* = 3.221E‐08, *n* = 25; Figure 1D,F), underscoring the idea that in a multispecies context, amplification bias is a relative (rather than absolute) phenomenon. The proportions of reads for the two markers that contained the largest number of mismatches (Ceph16S vs. MiFishU) were the least strongly correlated (τ = 0.51, *p* = 1.362E‐04, *n* = 25; Figure 1G).

### 3.2 Concentration of Total vs. Template DNA

#### 3.2.1 Template Concentration of Mock Species

**FIGURE 2:**
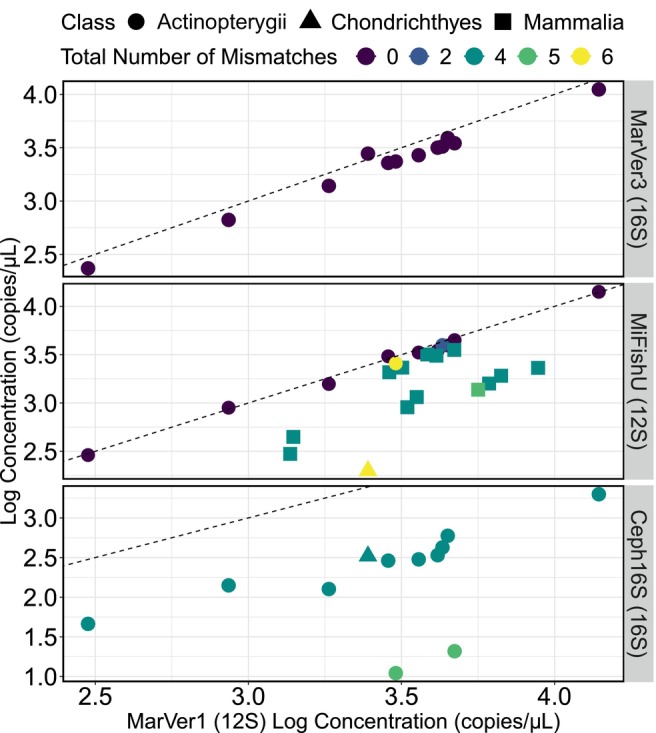
Comparison of measured log concentrations (copies/μL) via ddPCR for each species in the mock community. MarVer3 (16S), MiFishU (12S), and Ceph16S (16S) is compared against MarVer1 (12S), which contained no mismatches between the primer and species template. For species that contain mismatches between primer‐template for MiFishU, and Ceph16S, measured values fall below the 1:1 line, indicating ddPCR measurements of concentration are underestimated for those species.

For each species extract in the mock community, we quantified the concentrations (via ddPCR) of each template for each primer set (MarVer1 and MiFishU for all fishes and cetaceans; MarVer3 and Ceph16S for only fishes). For primer‐template pairs that contained no mismatches and zero or one degenerate base position (see next section), we found that estimates of the template concentration were nearly the same for species across all primer sets—both within a gene (e.g., MiFishU [12S] vs. MarVer1 [12S]) and across genes (e.g., MarVer1, MiFishU [12S] vs. MarVer3 [16S]), indicating that these are robust—and likely unbiased—estimates of the mtDNA concentrations in a sample for each species (Figure 2A,B). Conversely, for species with primer‐template mismatches, ddPCR systematically underestimated template concentrations (e.g., see Ceph16S vs. MarVer3 [16S] and MarVer1 [12S], Figure 2C).

Hereafter, we refer to “total gDNA concentrations” as those estimated via Qubit Fluorometry, and “template mtDNA concentrations” as those estimated by a ddPCR assay using the MarVer1 primer set, which contains no mismatches to any species in our mock community. We report the proportions of each species in the mock community calculated by each of these methods (Table 2). We found that the percentage of mtDNA to the total gDNA proportion varied across all species, with no apparent pattern to class (Table 2, Figure S5).

**TABLE 2:** A comparison of proportions of each species in the mock community using either concentration of genomic DNA (gDNA) via Qubit and concentrations of mitochondrial DNA (mtDNA) via ddPCR. The percentage of mtDNA to gDNA (in ng/μL) calculated from converting ddPCR copies/μL of MarVer1 (12S) to ng/μL and dividing by Qubit concentration (in ng/μL). *Ceratoscopelus townsendi* is omitted because it failed to amplify for 12S primer sets.

Species	Common name	Class	Proportion from gDNA	Proportion from mtDNA
*Carcharodon carcharias*	Great white shark	Chondrichthyes	0.0756	0.0450
*Clupea pallasii*	Herring	Actinopterygii	0.0756	0.2736
*Diogenichthys atlanticus*	Longfin lanternfish	Actinopterygii	0.0756	0.0845
*Engraulis mordax*	Northern anchovy	Actinopterygii	0.0756	0.0853
*Hippoglossus stenolepis*	Pacific halibut	Actinopterygii	0.0756	0.0310
*Leuroglossus stilbius*	California smoothtongue	Actinopterygii	0.0756	0.0916
*Merluccius productus*	North Pacific hake	Actinopterygii	0.0756	0.0607
*Oncorhynchus nerka*	Sockeye salmon	Actinopterygii	0.0756	0.0493
*Oncorhynchus tshawytscha*	Chinook salmon	Actinopterygii	0.0756	0.0050
*Sardinops sagax*	Pacific sardine	Actinopterygii	0.0756	0.0209
*Thaleichthys pacificus*	Eulachon	Actinopterygii	0.0756	0.0793
*Trachurus symmetricus*	Pacific jack mackerel	Actinopterygii	0.0756	0.0634
*Balaenoptera acutorostrata*	Minke whale	Mammalia	0.0756	0.0094
*Balaenoptera musculus*	Blue whale	Mammalia	0.0066	0.0064
*Balaenoptera physalus*	Fin whale	Mammalia	0.0066	0.0048
*Delphinus delphis*	Common dolphin	Mammalia	0.0066	0.0022
*Globicephala macrorhynchus*	Pilot whale	Mammalia	0.0066	0.0126
*Grampus griseus*	Risso's dolphin	Mammalia	0.0066	0.0021
*Megaptera novaeangliae*	Humpback whale	Mammalia	0.0066	0.0085
*Mesoplodon densirostris*	Blainville's beaked whale	Mammalia	0.0066	0.0067
*Orcinus orca*	Killer whale	Mammalia	0.0066	0.0122
*Peponocephala electra*	Melon headed whale	Mammalia	0.0066	0.0059
*Phocoena phocoena*	Harbour porpoise	Mammalia	0.0066	0.0140
*Phocoenoides dalli*	Dall's porpoise	Mammalia	0.0066	0.0066
*Physeter catodon*	Sperm whale	Mammalia	0.0066	0.0143
*Ziphius cavirostris*	Cuvier's beaked whale	Mammalia	0.0066	0.0047

#### 3.2.2 Calibration with Quantitative Metabarcoding Model

**FIGURE 3:**
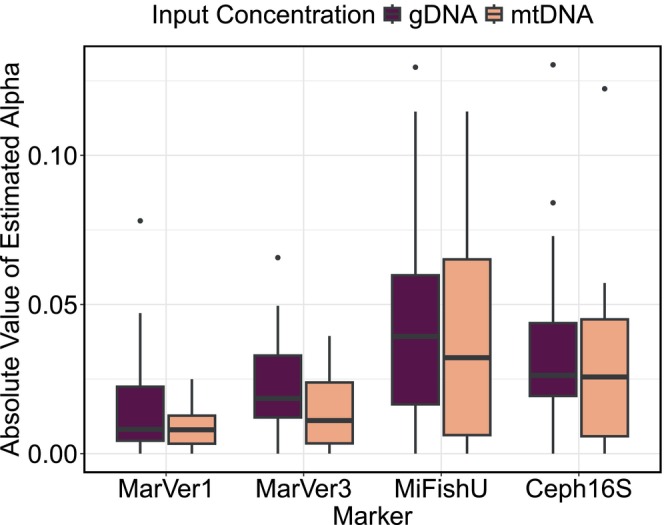
The absolute value of the estimated alpha α (amplification efficiency) for all species after calibrating proportions using the quantitative metabarcoding model (in Shelton et al. 2023) with total genomic DNA (gDNA) versus mitochondrial template DNA (mtDNA) for markers with perfect matches to all species in the mock community (MarVer1 [12S] and MarVer3 [16S]) and with imperfect matches for some species (MiFishU [12S] and Ceph16S [16S]). Note, the higher the absolute value of α, the more biased the measure of amplification efficiency.

Primer sets that contained perfect matches to all species in the mock community (MarVer1 and MarVer3) showed little bias, measured by smaller values of estimated amplification efficiencies (α; a measured in relation to the reference species *Engraulis mordax* ), when calibrated with template mtDNA versus total gDNA (Figure 3; average absolute value of α for MarVer1 and MarVer3, respectively: mtDNA = 0.0088 ± 0.0071 and 0.016 ± 0.13 vs. gDNA = 0.018 ± 0.019 and 0.022 ± 0.017). By contrast, for primer sets that contained mismatches to some species in the community (MiFishU and Ceph16S), bias was still large when calibrating with both mtDNA and gDNA (average absolute value of α for MiFishU and Ceph16S, respectively: mtDNA = 0.036 ± 0.033 and 0.028 ± 0.028 vs. gDNA = 0.042 ± 0.034 and 0.036 ± 0.029).

**FIGURE 4:**
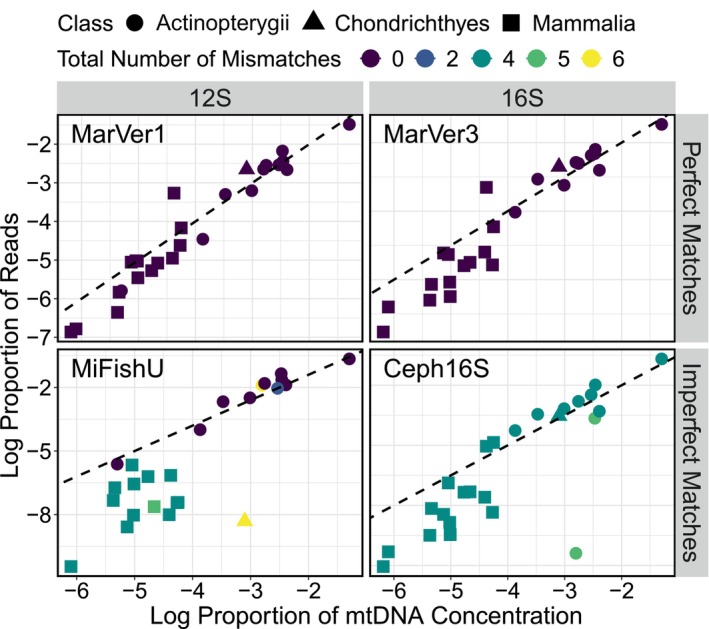
The log proportion of metabarcoding reads for each primer set in relation to the log proportion of concentration of mtDNA (as determined via ddPCR using MarVer1 for each species; treated as the true template concentration present), with class and total numbers of mismatches (summed across both forward and reverse primers) between primer‐template pairs indicated by shapes and colours, respectively. The dotted line represents the 1:1 line. Note that where some species are underrepresented in the metabarcoding data, other species are concomitantly over‐represented because proportions of reads must sum to one.

Most critically for the interpretability of metabarcoding data, in the absence of mismatches, the observed proportions of species' reads closely matched the proportions species' mtDNA template concentrations (MarVer1: τ = 0.86, *p* = 9.964E‐08, *n* = 25; MarVer3: τ = 0.82, *p* = 1.562E‐11, *n* = 25). By contrast, where mismatches were present, this correlation was weaker because those species with primer‐template mismatches were underrepresented relative to their true proportions and accordingly, those with perfect primer‐template matches were overrepresented (Figure 4; MiFishU: τ = 0.59, *p* = 1.093E‐05, *n* = 25; Ceph16S: τ = 0.69, p = 9.964E‐08, *n* = 25).

### 3.3 Distinguishing Causes of Amplification Bias

#### 3.3.1 Mismatches Between Primer‐Template

**FIGURE 5:**
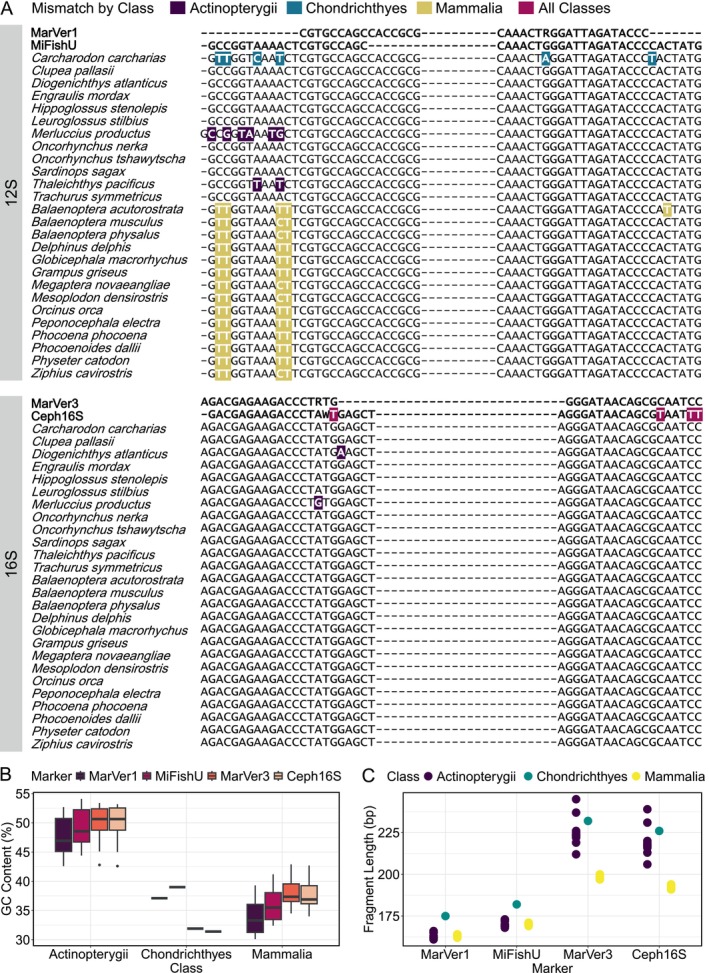
(A) Alignments of 12S (MarVer1 and MiFishU) and 16S (MarVer3 and Ceph16S) primer binding site regions for species in the mock community. Primer sequences are bolded, and the highlighted bases indicate the mismatch positions between the primer and species sequences, with the mismatches coloured by class. For primers containing degeneracies, a perfect match was considered as matching either base in the degenerate position (e.g., an A was a perfect match to an R [which is either an A or G]). (B) GC content of the amplicon (without the primer binding site) for each marker across class, coloured by Marker. (C) Fragment length of each amplicon (without the primer binding site) for each marker, coloured by class.

We generated alignments for 12S (MiFishU and MarVer1) and 16S (MarVer3 and Ceph16S) (Figure 5). We found that for all species in the mock community, MarVer1 and MarVer3 contained no mismatches between the primer and template in the forward and reverse primer binding region. The Ceph16S primer set contained a 1 and 3 bp mismatch in the forward and reverse primer, respectively, to all templates in the mock community; that is, all species shared the same number and position of mismatches for Ceph16S. *Merluccius productus* and *Diogenichthys atlanticus* contained an additional 1 bp mismatch to the Ceph16S forward primer.

The forward primer of MiFishU had mismatches with the following fish species: *Carcharodon carcharias* (4 bp), *Merluccius productus* (6 bp), and *Thaleichtys pacificus* (2 bp). The reverse primer of MiFishU had mismatches for only *Carcharodon carcharias* (2 bp). Although *Merluccius productus* had a 6 bp mismatch between the primer and template, this primer binding region contained an insertion, making the primer binding site offset from the primer by 1 bp on either end. All cetacean species had a 4 bp mismatch in the forward MiFishU primer and a perfect match to the reverse MiFishU primer, except for *Balaenoptera acutorostrata*, which contained a 1 bp mismatch in the reverse primer binding region. The mismatches in the forward primer binding region for cetaceans fell into two groups: (1) containing an AC‐TT primer‐template mismatch and (2) containing an AC‐CT primer‐template mismatch.

#### 3.3.2 GC Content and Fragment Length

Template sequences deriving from species in different taxonomic classes differed in GC content. Actinopterygii species amplicons had a higher GC content (approximately 45%–50%) compared to the Mammalia and Chondrichthyes species (approximately 35%–40%; Figure 5B) for all markers. There was also a difference in fragment length of amplicons between classes, which was more pronounced for the 16S markers (MarVer3. Ceph16S). Here, Actinopterygii and Chondrichthyes species amplicons were approximately 20 bp larger than the Mammalia species amplicons. Instead, for 12S, all classes had a similar size structure of amplicon length (Figure 5C).

#### 3.3.3 Modelling Amplification Bias

The best fitting model indicated that 64.2% of the observed amplification efficiency (α) could be explained by the interaction between Taq polymerase and the number of mismatches, the interaction between Taq polymerase and GC content of the amplicon, and the fragment length of the amplicon.

The number of mismatches had a negative effect on amplification efficiency (compared to the reference species *Engraulis mordax* ), and the extent of this effect varied with Taq. The Qiagen Multiplex Master Mix had the largest negative effect (mean = −2.64E‐02 ± 1.45E‐03, 95% confidence interval: −0.0274, −0.02394), followed by Invitrogen Platinum Superfi (mean = −1.79E‐02 ± 1.46E‐03, 95% credible interval: −0.02035, −0.01548), Promega GoTaq Fusion (mean = −1.53E‐02 ± 1.37E‐03, 95% credible interval: −0.01755, −0.01306), and finally NEB Phusion HiFi (mean = −1.06E‐02 ± 1.35E‐03, 95% credible interval: −0.01277, −0.09842).

The effect of GC content was minor but significant for only one of the four tested Taq polymerases (NEB Phusion HiFi), with a positive effect on amplification efficiency in relation to *Engraulis mordax* (mean = 8.49E‐04 ± 3.00E‐04, 95% credible interval: 0.00035, 0.000136). Fragment length also had a minor but significant positive effect in relation to *Engraulis mordax* (mean = 5.45E‐04 ± 8.20E‐05, 95% credible interval: 0.00041, 0.00068).

Effects for all tested predictors can be found in Appendix S1: Section S5.

#### 3.3.4 Assessing Bias in a Community Context Using Metabarcoding Data

**FIGURE 6:**
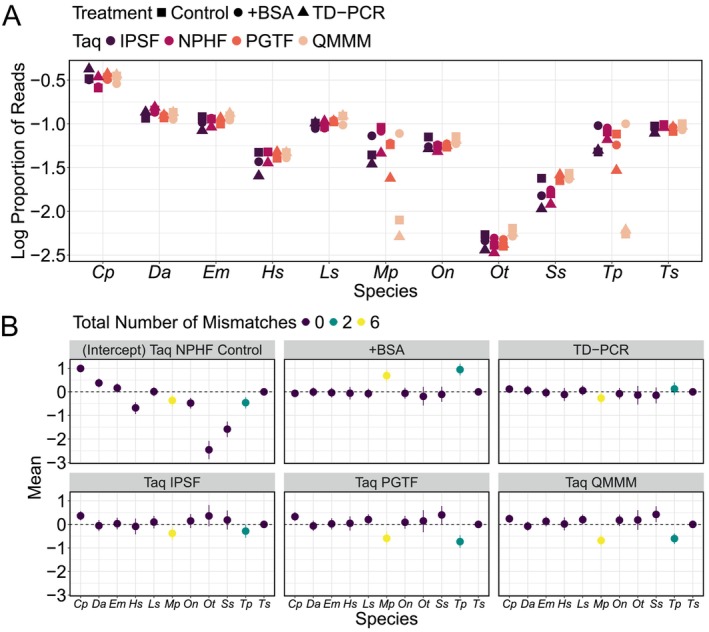
Differences in treatment on the proportion of reads for Actinopterygii species amplified with MiFishU, where treatments are control (no BSA, normal cycling conditions); +BSA (addition of BSA, normal cycling conditions); and TD‐PCR (no BSA, TD PCR) for each Taq polymerase. (A) Mean proportion of reads (from triplicate samples) for each treatment, for each Taq polymerase used (IPSF = Invitrogen Platinum SuperFi; NPHF = NEB Phusion HiFi; PGTF = Promega GoTaq Flexi; QMMM = Qiagen Multiplex Master Mix). (B) Fitted parameters from zoid by treatment, with the intercept term given for the control treatment with NPHF in relation to *Trachurus symmetricus* in the first panel; and the change in that term given for each treatment in the next panels (whiskers show 95% posterior credibility interval for each effect; values of effect sizes on y‐axis are in additive log‐ratio space relative to the reference condition), with colours denoting the total number of mismatches to the forward and reverse primers (summed). Species are as follows: *Clupea pallasii* (*Cp*), *Diogenichthys atlanticus* (*Da*), *Engraulis mordax* (*Em*), *Hippoglossus stenolepis* (*Hs*), *Leuroglossus stilbius* (*Ls*), *Merluccius productus* (*Mp*), *Oncorhynchus nerka* (*On*), *Oncorhynchus tshawytscha* (*Ot*), *Sardinops sagax* (*Ss*), *Thaleichthys pacificus* (*Tp*), and *Trachurus symmetricus* (*Ts*).

We examined the effect of varied PCR protocols on reads assigned to Actinopterygii species using MiFishU. Overall, we did not observe a large treatment effect on the proportion of reads across all Actinopterygii species (Figure 6; effect sizes in Figure 6B are at or near zero for most species in most treatments).

However, we observed some species‐specific effects, particularly for those species that contained mismatches to the primers (see Figure 6B for variation in treatment effects across species and treatments). For example, the addition of BSA resulted in a higher proportion of reads for both species that contained mismatches, *Merluccius productus* (mean = 0.689; 95% credible interval: 0.444, 0.936) and *Thaleichthys pacificus* (mean = 0.944; 95% credible interval: 0.698, 1.196; Figure 6B). There was no significant effect of TD‐PCR for any species. The effect of different Taqs was also species‐specific. When comparing to the reference Taq (NEB Phusion HiFi), there was a negative effect for species containing mismatches to the primer set (95% credible interval for *Merluccius productus* : −1.087, −0.448; and for *Thaleichthys pacificus* : −0.741, −0.123) and because the data is compositional and must sum to one, an associated positive effect on the most abundant species *Clupea pallasii* (95% credible interval: 0.171, 0.559). For the non‐high fidelity Taq polymerases (PGTF and QMMM), there were significant effects for the two mismatched species ( *Merluccius productus* and *Thaleichthys pacificus* ) and for the two species with perfect matches to the primer set ( *Clupea pallasii* and *Sardinops sagax* ). The mismatched species contained a lower proportion of reads compared to NEB Phusion HiFi (PGTF 95% credible intervals for *Merluccius productus* : −0.855, −0.318; and for *Thaleichthys pacificus* : −0.996, −0.459; and QMMM 95% credible intervals for *Merluccius productus* : −0.914, −0.450; and for *Thaleichthys pacificus* : −0.834, −0.384). By contrast, *Clupea pallasii* contained a significantly higher proportion of reads compared to NEB Phusion HiFi (PGTF 95% credible interval: 0.142, 0.519; QMMM 95% credible interval: 0.066, 0.412), as did *Sardinops sagax* (PGTF 95% credible interval: 0.023, 0.777; QMMM 95% credible interval: 0.094, 0.767). Model results can be found in Appendix S1: Section S6.

## 4 Discussion

Here, we constructed a mock community and amplified it with different primer sets and Taq polymerases to investigate processes driving amplification bias in metabarcoding studies. We first compared methods of calibration and found that calibration of expected proportions from template DNA concentrations, rather than total genomic DNA, was most appropriate. By doing the former, we were able to more accurately disentangle observation bias derived from differing ratios of mtDNA to gDNA template in the extract from observation bias derived from amplification processes. After calibrating our data using expected proportions based on mtDNA concentrations, we were able to explain more than 60% of amplification bias by characteristics of the DNA of the mock community members. In our dataset, we found that most of the bias was driven by mismatches between the primers and template (to different extents depending on Taq polymerase); fragment length was also important in explaining bias, and GC content was important for one Taq polymerase. Our results showed that in a community context, the effects of PCR protocols on the proportion of reads were species‐specific and most variability between PCR treatments occurred for species that contained mismatches to the primer binding site. We can leverage these insights to understand the community composition from which metabarcoding data arise and quantify biases more accurately, which can ultimately allow metabarcoding analyses to move towards quantitative inference (although the link between concentration of eDNA and organism abundance/biomass remains an important frontier). This extension is key for users not only in ecological fields that rely on accurate estimates of species abundance (e.g., assessments for fisheries, endangered species, biodiversity, etc.), but also in core biomedical applications such as transcriptomics and microbiome analyses.

### 4.1 DNA Concentration in Relation to Observation Bias: Amplicon Proportions Reflect Amplifiable Template

In general, models built to calibrate metabarcoding data measure observation bias by first constructing a mock community of known composition and then correcting observed read proportions to counteract that observation bias (McLaren et al. 2019; Shelton et al. 2023; Silverman et al. 2021). Different approaches to estimating the expected (“known”) proportions of DNA before amplification can yield significant differences in those estimates and thus result in significant differences in the resulting estimate of observation bias. Here, we considered two ways to quantify the proportion of each species before PCR amplification: (1) using total genomic DNA concentration and (2) using mtDNA concentration as measured by ddPCR. We found that for species that contained perfect matches to the primer (as in the case with MarVer1 and MarVer3), after correcting proportions based on mtDNA template concentrations, there was a 51% decrease in amplification bias for species amplified with MarVer1 (average absolute value of α from 0.018 with gDNA to 0.0088 with mtDNA) and a 27% decrease in amplification bias with MarVer3 (average absolute value of α from 0.022 with gDNA to 0.016 with mtDNA). However, for species that contained mismatches to the primer set (as in the case of MiFishU and Ceph16S), even after correcting for template concentrations, there was still a considerable amount of amplification bias observed (e.g., percent decrease of only 14% for MiFishU of 22% and for Ceph16S, with all mean values for absolute value of α for both gDNA and mtDNA greater than 0.028).

For eDNA studies examining macroinvertebrate and vertebrate taxa, regions of the mitochondrial genome are typically targeted for amplification because more comprehensive reference sequences are available for mtDNA markers. Different tissue types with different cellular functions can have varying abundances of mitochondria, which can change with life stage and can be subject to phenotypic plasticity (Calogero et al. 2023; Hartmann et al. 2011; Liu et al. 2018; Veltri et al. 1990). Thus, when constructing a mock community based on total genomic DNA of extracts, additional observation bias arises because of the varying ratios of template to total DNA between extracts. Overall, we found that mtDNA made up 0.005% or less of the total genomic DNA in all species extracts (Figure S5), where we assumed that nontarget DNA was minimal and consistent for all pure DNA extracts from which we derived total genomic DNA with Qubit. The ratio was also not consistent across species (Figure S5), further highlighting the hidden observation bias across extracts (and presumably tissue types) when considering expected proportions from total genomic DNA.

While our finding that amplicons more closely relate to expected proportions based on template concentrations rather than total genomic DNA is not new, and perhaps intuitive, thinking in terms of total gDNA may make more ecological sense when relating eDNA back to species biomass, abundance, and so forth. For instance, a higher mtDNA template concentration for a given species relative to other species in the mixture may not necessarily translate to a higher biomass of that species. As we begin to understand the type of biological material captured in eDNA sampling (e.g., whether cellular, organellar, or extracellular; Kirtane et al. 2023; Mauvisseau et al. 2022; Powers et al. 2023), and how variable it is, we may be able to gain more insight from mtDNA:gDNA ratios, and understanding these ratios in different life stages and tissue types, and between different species, may be crucial in linking metabarcoding data back to true organism abundance, biomass, and so forth in a given system.

### 4.2 Compositional Data Complicates Interpretation of Proportions

The binding affinity of a primer‐template for a given species is a function of thermodynamics (SantaLucia and Hicks 2004; Stadhouders et al. 2010)—and therefore it remains consistent whether in isolation (as in qPCR/ddPCR) or in a multispecies context. However, metabarcoding observations arise in a multispecies mixture in which template molecules compete for reagents and for sequencing read depth. Including any low‐affinity primer‐template set (e.g., that contains mismatches) will result in the affected taxa being underrepresented, and because metabarcoding data are compositional, observed proportions of other taxa in the dataset will be inflated relative to their underlying template proportions. Thus, whenever low‐affinity primer‐template pairs are present in a metabarcoding dataset (which is often the case for eDNA metabarcoding studies), the proportional estimates of all taxa are unreliable until appropriately calibrated. Note that this applies not only to metabarcoding studies, but to any kind of PCR‐based multi‐taxon study, such as those common in microbial ecology and medicine. One solution to this problem is to subset the overall dataset to focus solely on species of interest (see Shelton et al. 2023) for which primer mismatches and other relevant information are known.

### 4.3 Species‐Specific DNA Characteristics Drive Amplification Bias in Metabarcoding Data

#### 4.3.1 Mismatches Cause Species to Be Underrepresented Proportionally

The most pronounced effect on amplification bias was mismatches between the primer‐template, where primer‐template sets with mismatches were poor amplifiers or, in extreme cases, non‐amplifiers (e.g., Mammalia species that contained four mismatches to the primer set had low to no read abundance when amplified with MiFishU). Interestingly, the magnitude of the effect of mismatches varied between Taq polymerases. When the mock community was amplified with the Qiagen Multiplex Master Mix, mismatches had the greatest negative effect on amplification efficiency (and the associated proportion of reads) compared to the other Taq polymerases. NEB Phusion HiFi had the least pronounced (but still) negative effect of mismatches. The former polymerase is a hot‐start polymerase that does not have high fidelity nor extra proofreading capability, whereas the latter is a non‐hot‐start, high fidelity polymerase with a proofreading enzyme. Characteristics of different Taq polymerases cause them to function differently during annealing and elongation. They have different error rates associated with PCR, and performance has been shown to vary with mismatch type and position (Kwok et al. 1990; Eckert and Kunkel 1991; McInerney et al. 2014; Rejali et al. 2018; Stadhouders et al. 2010). Because the effect of Taq was most pronounced for species with mismatches, and eDNA samples typically contain a mixture of taxa with varying levels of mismatch to the primer binding site, it is most appropriate when correcting unknown eDNA samples with a mock community to use the same marker‐Taq combination. This point also supports the idea that primer sets that are more highly targeted to a specific taxonomic group may behave more predictably across different Taq polymerases.

We find here that even when the proportion of reads from metabarcoding data closely resembles expected proportions based on mtDNA concentrations, species may not be necessarily amplifying at 100% efficiency. For instance, we found that when the mock community was amplified with Ceph16S, in which almost all fish and cetacean species contained the same type and position of mismatches to the primers, read proportions appeared proportionally unbiased and closely reflected the template proportions (τ = 0.69, *p* = 9.964E‐08, *n* = 25; Figure 4D)—even though measurements of Ceph16S template via ddPCR were all underestimated (see Figure 2C). This is an important consideration when using a subset of metabarcoding data, as a subset of a group of organisms that contain similar DNA characteristics (e.g., mismatches, GC content and fragment length) yields more unbiased data. Another consideration when analysing a subset of species from a sample is considering the proportion of the total reads that were assigned to the species of interest in the subset; for instance, if the subsampled community is a smaller proportion of the total reads (e.g., in our case for Ceph16S, where fish and cetaceans only made up 30% of the total reads), random variability due to low read abundance (and/or lower DNA concentration) may be of greater relative importance and result in more stochastic data (which would likely be reflected in more variable technical replicates).

#### 4.3.2 Degeneracies Could Impact Proportions Even if “Perfect Match” to Primer

The quantitative metabarcoding model in Shelton et al. (2023) assumes that species‐specific amplification is independent of other species in the mixture, and so amplification efficiencies are repeatable for a primer‐species pair (or as we show here, a primer‐Taq‐species combination). It also assumes that there is no competition for the primer, for example, that there is only one primer present acting upon the community. However, in eDNA studies, we often employ degenerate primers to broaden the taxa that we can amplify (e.g., the commonly used Leray COI primers [Leray et al. 2013] capture high metazoan diversity by having a degenerate nucleotide in every third position). A primer containing any number of degeneracies is effectively a mix of a large number of unique primers—the number is a factorial expansion of the unique combinations of degeneracies present—and each of these primers amplifies a given template molecule at a different amplification efficiency.

Accordingly, when degenerate primer sets are used, predicting proportions of species is likely to be far more challenging. In our dataset, we have two instances of perfect match primers containing a degeneracy of two possibilities of bases, and all but one member of the mock community match one of the two bases. For MarVer1‐R, the seventh position contains a degenerate R, and all species in the mock community have an A at this position except *Carcharodon carcharias* , which contains a G; for MarVer3‐F, the 16th position contains a degenerate R, and all species have an A except *Merluccius productus* , which contains a G. In these two cases, the species present in the mock community in reality have a separate amplification efficiency for each of the two primers present, and so it is perhaps more appropriate to predict biases for the communities with two separate primer sets, resulting in two amplification efficiencies per species per marker. Instead, the amplification efficiencies we report here have these differential amplification efficiencies wrapped up in one term, but more accurate estimates of amplification efficiency accounting for degeneracies may allow us to predict and explain bias to a greater degree, and future investigation is warranted here.

#### 4.3.3 When All Species Perfectly Match Primers, Bias Still Present

In perfect match communities, there were still biases present due to amplification processes after correcting for mtDNA template concentration, and such biases were greater in MarVer3 than MarVer1. MarVer1 contained a more homogenous mixture in terms of DNA characteristics versus MarVer3, in which differences in fragment length were more pronounced. This is consistent with our findings from modelling amplification bias for the skewed community (see Section 5.3). This skewed community dataset only contained information from the 12S markers (MarVer1 and MiFishU) and the best fit model that explained amplification bias contained only mismatches and GC content, and instead fragment length was not important (intuitively, as the 12S amplicons were homogenous in fragment length).

The effect of GC content was only significant for one Taq polymerase (NEB Phusion HiFi), and this was also supported in the analysis of the skewed community (see Appendix S1: Section S5.3). In a previous study examining Taq polymerase performance in relation to GC content, Taq polymerases were shown to prefer certain GC contents, and NEB Phusion HiFi exhibited the most GC bias (Nichols et al. 2018). Taqs perform optimally within a specific GC range, and so GC bias across Taqs may depend on the makeup of GC contents of the species in the community being amplified. It is apparent that bias is decreased if the community contains similar DNA characteristics (in that they are all not biased or equally biased, which both yield similar read proportions)—again a case for using highly targeted primers that target a narrow taxonomic group with similar DNA characteristics as being most useful quantitatively interpreting metabarcoding data.

The factors we examined here only explained roughly 60% of amplification bias derived from PCR and sequencing. Other factors that may influence amplification bias are more difficult to measure and predict, such as the DNA structure of longer folded strands and the associated complexity (or base stacking), which is ultimately dictated by the order of base pairs (Chen and Skylaris 2021) and thermodynamics (SantaLucia and Hicks 2004). Hairpins in DNA strands form when pieces of the strand bind to one another to form loops and can affect PCR efficiency (Singh et al. 2000). Similar to the predictors we explored here, these factors are inherent to a template sequence (and thus to a given haplotype or putatively to a species), and may act together to influence the final metabarcoding output to be biased towards or against a particular species and should also be considered when trying to measure observation bias.

### 4.4 Different PCR Protocols Can Cause Changes in Community Composition due to Species‐Specific Effects

We found that for MiFishU, changing the Taq polymerase or adding BSA mainly affected species that contained mismatches to the primers ( *Merluccius productus* and *Thaleichthys pacificus* ) in relation to the reference condition of the community amplified with NEB Phusion HiFi with no BSA and normal cycling conditions. The effect of Taq on the proportion of reads in our community composition analysis (e.g., the zoid model) is consistent with our findings from examining our model that showed Taq interacted with mismatches to influence amplification bias (e.g., the stan_lm model).

BSA increased the proportions of both species with mismatches to the primers in our dataset. BSA is an adjuvant and has been shown to increase PCR yields of low purity template and decrease the effect of inhibitors on PCR (Kreader 1996; Nagai et al. 2008). While mismatches can be thought of as a type of inhibition, it remains unclear how BSA interacts with primer‐template mismatches and/or Taq to increase their amplification efficiency. TD cycling is typically used to increase primer specificity, but here we observed no effect of TD cycling for any fish species. This result may be misleading in the context of eDNA samples, which often contain a high ratio of off‐target species with more variation in mismatches to the primer site compared to a mock community. While TD cycling in these cases may decrease unwanted off‐target abundance, our results here indicate that it may not substantially alter read proportion and/or the amplification efficiencies of the species within the main target taxa of interest. That is, TD cycling may be more important for eliminating off‐targets that are more phylogenetically unrelated than what we present here in our mock community (e.g., the difference between bacteria and fish may be more pronounced compared to difference within fish).

### 4.5 Moving Out of Proportions and Into Absolute Quantification

After correcting for observation biases, it is also possible to move from proportional estimates to estimates of absolute quantity of template DNA. In some examples, calibrated proportional data from metabarcoding can be paired with qPCR/ddPCR assays of a reference species (see Andruszkiewicz Allan et al. 2023). Some other studies have suggested incorporating a DNA standard (or spike‐in) during eDNA metabarcoding to aid in quantifying DNA abundance (Sato et al. 2021; Stoeckle et al. 2022; Tsuji et al. 2020; Ushio et al. 2018; Zemb et al. 2020). With this method, by knowing the starting concentration of one proportion (i.e., the standard) before PCR amplification and the proportion of reads assigned to the standard after metabarcoding, one can determine the starting concentrations for all other proportions (and thus each species). However, our findings suggest calibration via a spike‐in warrants some considerations. It appears that a spike‐in would only be valid (1) for those species that have perfect matches to the primer, (2) if no species with mismatches were simultaneously included in the analysis, and (3) if the spike‐in also has perfect matches to the primer. When there is an inexact match between primer and template, species with mismatches will be underrepresented compared to their actual starting concentrations—and because species‐read proportions must sum to one within a sample, all species without mismatches will be accordingly overrepresented. Even after all species in the community and the spike‐in contain perfect matches, we found evidence that bias still occurs due to fragment length and for some Taq polymerases, GC content. Therefore, to minimise such bias, the spike‐in should contain similar characteristics to that of the target taxa (which may not be possible for markers that amplify a wide array of taxa). Furthermore, while we did not investigate the structural complexity of the longer DNA strand (see Chen and Skylaris 2021), it could be considered and further investigated how the standard (which presumably is a shorter synthetic strand of DNA) amplifies compared to a longer, more complex DNA strands.

## 5 Conclusion

Observed sequence proportions in metabarcoding studies differ—often substantially—from the proportions of DNA present in mock communities. We find that, in the absence of mismatches, observed proportions closely correlate to expected proportions based on template concentration. However, this correlation erodes in communities with species that do not bind with 100% efficiency to the primer set (e.g., contain mismatches between primer‐template). Primer choice therefore largely drives the observed metabarcoding output. If primers are a perfect match to all members of the community, bias is reduced substantially but is not eliminated entirely; other DNA characteristics including fragment length (and in some instances GC content) explain some of the residual bias. Using primers that target a specific community with members that contain similar DNA composition (e.g., one taxonomic group) is therefore likely to yield datasets more immediately useful for quantitative analysis. Our data demonstrate that we can measure, and to some extent predict, observation bias in metabarcoding, which is a critical step in making this method more quantitative and robust.

## Author Contributions

Designed research: M.R.S., E.A.A., A.M.V.C., K.M.P., R.P.K. Acquired funds and resources: M.R.S., K.M.P., R.P.K. Performed research: M.R.S., R.P.K. Analysed data: M.R.S., E.A.A., A.M.V.C., R.P.K. Wrote the paper: M.R.S., R.P.K. Reviewed and edited paper: M.R.S., E.A.A., A.M.V.C., K.M.P., A.O.S., R.P.K.

## Conflicts of Interest

The authors declare no conflicts of interest.

## Supporting information


Appendix S1.

## Data Availability

Data and associated code can be found here: https://zenodo.org/records/15020661 (Shaffer et al. 2024).
