# Supplementary material for: Observation Bias in Metabarcoding
Source: Mol Ecol Resour. 2025 May 15;25(7):e14119. doi: 10.1111/1755-0998.14119 (PMC12415808; doi:10.1111/1755-0998.14119)
Supplement: Supplementary file 1 — Appendix S1. [file MEN-25-e14119-s001.pdf]

Supplement. Observation Bias in Metabarcoding

Megan R. Shaffer[1,2], Elizabeth Andruszkiewicz Allan[1], Amy M. Van Cise[3], Kim M. Parsons[2], Andrew Olaf Shelton[2], Ryan P. Kelly[1]

[1]School of Marine and Environmental Affairs, University of Washington, Seattle, Washington, USA
[2]Conservation Biology Division, Northwest Fisheries Science Center, National Marine Fisheries Service, National Oceanic and Atmospheric Administration, Seattle, Washington, USA
[3]School of Aquatic and Fishery Sciences, University of Washington, Seattle, Washington, USA

S1. Mock Community Information

In the following lab protocols that we describe here, we minimized contamination by using separate labs designated for pre-PCR (no DNA), extractions (low DNA) and post-PCR (high DNA/amplicons). For all lab work, the work benches were cleaned with DNA AWAY Surface Decontaminant and/or 10% bleach, followed by 70% ethanol, before and after all lab work. We UVed all pipettes and consumables (e.g., plates, tips, tubes, etc.) for 15 min before use. To track potential contamination, we added extraction blanks during DNA extraction and no template controls during PCR.

S1.1. Sample Information and Mock Community Construction

We constructed the mock community using tissue extracts from 36 species found in Table S1, which are all common to the US West Coast. Our mock community consisted of species belonging to the Classes Actinopterygii (N = 12), Chondrichthyes (N = 1), Malacostraca (N = 3), Cephalopoda (N = 6) and Mammalia (N = 14).

We acquired tissue extracts from fish from three sources: (1) from the Scripps Institution of Oceanography Marine Vertebrate Collection at the University of California San Diego (more information in Gold et al., 2020); (2) from the University of Washington (UW) Fish Collection (a collection shared between the UW School of Aquatic and Fisheries Sciences and the Burke Museum); and (3) from NOAA-Northwest Fisheries Science Center (NWFSC). Voucher information, if available, can be found in Table S1. For cetaceans, we acquired extracts from either NOAA-NWFSC or NOAA-Southwest Fisheries Science Center. We obtained krill extracts from the NOAA-NWFSC. Lastly, we obtained cephalopod specimens that were collected by NOAA-Fishery Resource Analysis and Monitoring Division from the 2021 West Coast Groundfish Bottom Trawl Survey. DNA from Scripps Institution of Oceanography Marine Vertebrate Collection was extracted using a Chelex 100 extraction method (Walsh et al., 1991), as described in Gold et al. (2020). DNA from the University of Washington (UW) Fish Collection and NOAA were extracted from either muscle or fin clips using a Qiagen DNeasy Blood and Tissue Kit according to the manufacturer's protocol, either by hand or using a Qiagen HT automated extraction robot.

To construct the mock community, we measured the total DNA concentration of each sample in triplicate using Qubit Fluorometer (Invitrogen) using the dsDNA high Sensitivity Assay Kit. We constructed two mock communities, which we call (1) the even mock community and (2) the skewed mock community (Figure S1).

Table S1. Sample information for species in the mock community. Voucher number abbreviations are as follows: SIO = Scripps Institute of Oceanography; UWFC = University of Washington Fish Collection; NWFSC = Northwest Fisheries Science Center (NOAA); SWFSC = Southwest Fisheries Science Center (NOAA).

Species	Common Name	Class	Voucher Number and Sample Information
Carcharodon carcharias	Great white shark	Chondrichthyes	SIO 04-43
Ceratoscopelus townsendi	Dogtooth lampfish	Actinopterygii	SIO 93-298
Clupea pallasii	Herring	Actinopterygii	NOAA-NWFSC uncategorized; Eastern North Pacific morph
Diogenichthys atlanticus	Longfin lanternfish	Actinopterygii	SIO 10-166
Engraulis mordax	Northern anchovy	Actinopterygii	SIO 05-79
Hippoglossus stenolepis	Pacific halibut	Actinopterygii	NOAA-NWFSC uncategorized; Eastern North Pacific morph
Leuroglossus stilbius	California smoothtongue	Actinopterygii	SIO 08-31
Merluccius productus	North Pacific hake	Actinopterygii	SIO 06-262
Oncorhynchus nerka	Sockeye salmon	Actinopterygii	UWFC 0156199
Oncorhynchus tshawytscha	Chinook salmon	Actinopterygii	UWFC 0201253
Sardinops sagax	Pacific sardine	Actinopterygii	SIO 04-102
Thaleichthys pacificus	Eulachon	Actinopterygii	NOAA-NWFSC 51985.2
Trachurus symmetricus	Pacific jack mackerel	Actinopterygii	SIO 03-75
Balaenoptera acutorostrata	Minke whale	Mammalia	NOAA-NWFSC unknown; Eastern North Pacific morph
Balaenoptera musculus	Blue whale	Mammalia	NOAA-SWFSC 195768
Balaenoptera physalus	Fin whale	Mammalia	NOAA-SWFSC 195698
Delphinus delphis	Common dolphin	Mammalia	NOAA-SWFSC 145268
Globicephala macrorhynchus	Pilot whale	Mammalia	NOAA-SWFSC 143674
Grampus griseus	Risso’s dolphin	Mammalia	NOAA-SWFSC 185553
Megaptera novaeangliae	Humpback whale	Mammalia	NOAA-NWFSC unknown; Eastern North Pacific morph
Mesoplodon densirostris	Blainville's beaked whale	Mammalia	NOAA-SWFSC l112680
Orcinus orca	Killer whale	Mammalia	NOAA-NWFSC unknown; Eastern North Pacific morph (transient)
Peponocephala electra	Melon headed whale	Mammalia	NOAA-SWFSC 118400
Phocoena phocoena	Harbor porpoise	Mammalia	NOAA-NWFSC unknown; Eastern North Pacific morph
Phocoenoides dalli	Dall's porpoise	Mammalia	NOAA-NWFSC unknown; Eastern North Pacific morph
Physeter catodon	Sperm whale	Mammalia	NOAA-SWFSC 118400
Ziphius cavirostris	Cuvier's beaked whale	Mammalia	NOAA-SWFSC 94598
Euphausia pacifica	North Pacific krill	Malacostraca	NOAA-NWFSC krill #6<br>S of San Francisco on Shimada 2021 hake survey<br>Collected by Alicia Billings

Species	Common Name	Class	Voucher Number and Sample Information
Thysanoessa spinifera	Krill	Malacostraca	NOAA-NWFSC krill #18<br>Newport<br>Collected by Kym Jacobson & Samantha Zeman
Nematoscelis difficilis	Krill	Malacostraca	NOAA-NWFSC krill #44<br>Location: 32.5121667, -118.33087<br>Expedition CCE-LTER, MOCNESS, 0-1054 m<br>Scripps (contact: Moira Decima, Lynsey Salas)
Cranchiidae sp.	Glass squid	Cephalopoda	NOAA-NWFSC Forensics ID 72000<br>2021 West Coast Grountrawl Survey
Gonatus onyx	Clawed armhook squid	Cephalopoda	NOAA-NWFSC Forensics ID 72001<br>2021 West Coast Grountrawl Survey
Histioteuthis heteropsis	Strawberry squid	Cephalopoda	NOAA-NWFSC Forensics ID 72002<br>2021 West Coast Grountrawl Survey
Loligo opalescens	Opalescent inshore squid	Cephalopoda	NOAA-NWFSC Forensics ID 72006<br>2021 West Coast Grountrawl Survey
Octopoteuthis deletron	Octopus squid	Cephalopoda	NOAA-NWFSC Forensics ID 72008<br>2021 West Coast Grountrawl Survey
Onykia robusta	Robust clubhook squid	Cephalopoda	NOAA-NWFSC Forensics ID 72009<br>2021 West Coast Grountrawl Survey

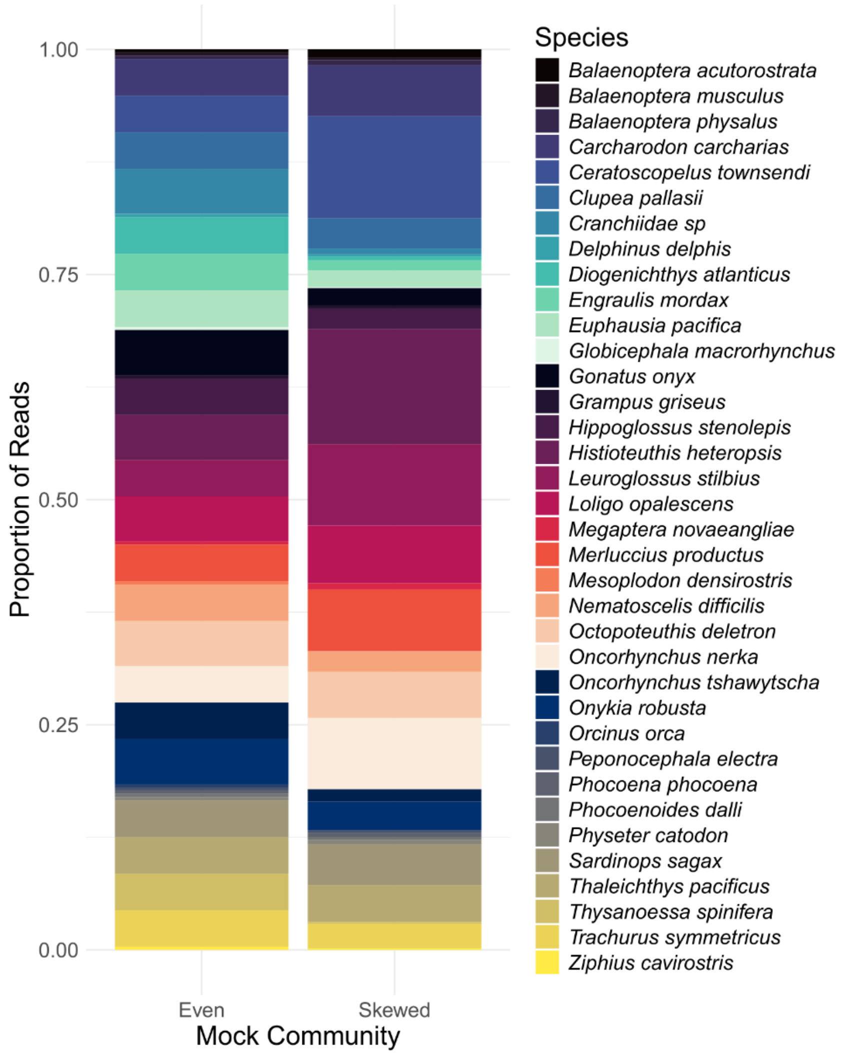

Figure S1. Expected proportions of reads for the even and skewed mock communities based on construction using genomic DNA concentrations.

To construct the even mock community, we created equimolar pools by combining: all fish and krill DNA extracts at equal gDNA concentrations; all cephalopod DNA extracts at equal gDNA concentrations; and all cetacean DNA extracts at equal gDNA concentrations. We then combined these equal concentration pools at different ratios to make the even mock community, wherein fish and krill made up 65% of the mock community, cephalopods made up 30%, and cetaceans the remaining 5% (Table S2, Figure S1).

To construct the skewed mock community, we created three pools again for fish and krill, cephalopods and cetaceans, but this time species within each mixture were at different percentages. We then combined these pools containing mixed concentrations to make the skewed mock community, wherein fish and krill made up 65% of the mock community, cephalopods made up 30%, and cetaceans the remaining 5% (Table S2, Figure S1).

Table S2. Proportions of species in the mock community based on total genomic DNA (via Qubit).

Species	Class	Even Proportion	Skewed Proportion
Balaenoptera acutorostrata	Mammalia	0.003571	0.009058
Balaenoptera musculus	Mammalia	0.003571	0.002899
Balaenoptera physalus	Mammalia	0.003571	0.005435
Delphinus delphis	Mammalia	0.003571	0.002174
Globicephala macrorhynchus	Mammalia	0.003571	0.001449
Grampus griseus	Mammalia	0.003571	0.003623
Megaptera novaeangliae	Mammalia	0.003571	0.007246
Mesoplodon densirostris	Mammalia	0.003571	0.000725
Orcinus orca	Mammalia	0.003571	0.000000
Peponocephala electra	Mammalia	0.003571	0.002536
Phocoena phocoena	Mammalia	0.003571	0.004710
Phocoenoides dalli	Mammalia	0.003571	0.003261
Physeter macrocephalus	Mammalia	0.003571	0.005072
Ziphius cavirostris	Mammalia	0.003571	0.001812
Ceratoscopelus townsendi	Actinopterygii	0.040625	0.113240
Clupea pallasii	Actinopterygii	0.040625	0.033972
Diogenichthys atlanticus	Actinopterygii	0.040625	0.004530
Engraulis mordax	Actinopterygii	0.040625	0.011324
Hippoglossus stenolepis	Actinopterygii	0.040625	0.022648
Leuroglossus stilbius	Actinopterygii	0.040625	0.090592
Merluccius productus	Actinopterygii	0.040625	0.067944
Oncorhynchus nerka	Actinopterygii	0.040625	0.079268
Oncorhynchus tshawytscha	Actinopterygii	0.040625	0.013589
Sardinops sagax	Actinopterygii	0.040625	0.045296
Thaleichthys pacificus	Actinopterygii	0.040625	0.040767
Trachurus symmetricus	Actinopterygii	0.040625	0.027178
Carcharodon carcharias	Chondrichthyes	0.040625	0.056620
Euphausia pacifica	Malacostraca	0.040625	0.018118
Nematoscelis difficilis	Malacostraca	0.040625	0.022648
Thysanoessa spinifera	Malacostraca	0.040625	0.002265
Cranchiidae sp.	Cephalopoda	0.050000	0.006383
Gonatus onyx	Cephalopoda	0.050000	0.019149
Histioteuthis heteropsis	Cephalopoda	0.050000	0.127660
Loligo opalescens	Cephalopoda	0.050000	0.063830
Octopoteuthis deletron	Cephalopoda	0.050000	0.051064
Onykia robusta	Cephalopoda	0.050000	0.031915

In the main paper, we analyse a subset the even mock community which contains only 26 species, including all cetaceans and fishes, except *Ceratoscopelus townsendi* because it had a poor quality extract that failed to amplify with any marker or with Sanger sequencing. This resulted in a subset of 14 cetaceans and 12 fishes. We excluded the krill and the cephalopods from our main analyses because the species identification of each specimen was not confirmed and because those supposed species had a few or no reference sequences available to allow species confirmation with Sanger sequencing. Because we were trying to understand the mechanisms behind observation bias, we removed these to reduce noise in the dataset. However, we report the composition of the entire mock community here in the Supplement for

those wanting to explore the dataset further now, or later when more reference sequences become available (Table S2, Figure S1).

S1.2. Species Confirmation of Mock Community Extracts for Subset of Species

To confirm the identity of the species we used in the main paper, we Sanger sequenced the subset of 26 species in the mock community. Sanger sequencing data does not capture intragenomic variants in mock members, and thus does not allow downstream differentiation of true intragenomic variants from spurious OTUs or ASVs that may arise from chimera formation during PCR and sequencing or from clustering algorithm errors (Jusino et al, 2019); however, we considered this method sufficient as a preliminary assessment to confirm that extracts were in fact the intended species during mock construction. We chose vouchered extracts when possible that have been previously deposited on GenBank (Table S3); for those extracts that were not previously deposited on GenBank, we confirmed species identity by comparing the Sanger sequence to reference sequences on GenBank via BLAST (Altschul et al., 1990).

For the fish extracts, we sequenced a 612 bp fragment that nests the MiFish 12S region using primers from Collins et al. (2021) (Aa22-PheF: AGCATAACACTGAAGATRYTARGA; Aa633-12sR: TTCTAGAACAGGCTCCTCTAG). We deposited the amplicon sequences onto GenBank for most species. There were some species that already had their 12S MiFish fragment deposited, but we still deposited the longer fragment so that the MiFishU primer binding sites could be made available. We recorded the accession numbers on the old GenBank submissions so that they can be linked to the previous submissions easily (Table S3).

For the cetacean extracts, we sequenced a ~450 bp fragment that contains the D-loop region, using primers H16498 (CCTGAAGTAAGAACCAGATG in Rosel et al. [1994] and LI5812 (CCTCCCTAAGACTCAAGG in Archer et al. [2013]). We chose D-loop as it is more variable and therefore provides more resolution for cetaceans than 12S or 16S. We submitted sequences for species for which we had voucher information. For a handful of the cetaceans, the voucher information was unknown due to lack of labelling of DNA aliquots, and so these sequences were not deposited on Genbank to avoid duplicate entries. Instead, these sequences can be found in the provided data files on Zenodo, in the file titled 'mammals_sanger_submit_genbank_CR_plus_tRNA-Pro.fasta'.

We amplified all extracts in 25 µL reactions with final concentrations of: 1X GoTaq Flexi Buffer (Promega), 3 mM of $MgCl_2$, 0.1 ug/µL BSA, 0.2 mM of each dNTP, 0.2 uM of the forward and reverse primers (listed above), and 1.25 u of GoTaq DNA Polymerase (Promega); with 2 µL of DNA extract, and the remaining volume with nuclease-free water. Thermocycling conditions were as follows: initial denaturation at 95°C for 2 min; followed by 35 cycles of denaturation (95°C for 30 sec), annealing (52°C for 30 sec), extension (72°C for 1 min); ending with a final extension at 72°C for 10 min. We visualized products on an agarose gel and cleaned products using a Millipore $MultiScreen_{96}$ PCR plate and vacuum. We then performed the sequencing reaction using the BigDye Terminator v3.1 Cycle Sequencing Kit (Applied Biosystems) for both forward and reverse strands, with the following recipe (per 10 µL rxn): 2 µL of 5X BigDye Terminator Sequencing Buffer, 1 µL of BigDye Terminator v3.1 Ready Reaction Mix, 0.32 µLof the forward or reverse 10 µM primer (depending on the strand sequenced), 2 µL of PCR product and the rest of the volume with nuclease-free water. We performed the BigDye Terminator reaction using the following cycling conditions: initial denaturation at 96°C for 1 min; followed by 30 cycles of denaturation (96°C for 10 sec), annealing (50°C for 5 sec), extension

(60°C for 4 min). We then cleaned the products using Agencourt CleanSeq beads (1X beads, followed by two 85% ethanol washes before elution). We Sanger sequenced the forward and reverse direction of each extract on 3500 Genetic Analyzer (Applied Biosystems) at NOAA Northwest Fisheries Science Center.

We used Geneious Prime to edit and build consensus sequences using both forward and reverse sequences for each extract. We then compared our Sanger sequences with those on NCBI's nucleotide database using BLAST (Altschul et al., 1990). Note some species produced no high-quality sequences after Sanger, likely due to either poor primer affinity or poor extract quality (Table S3).

Table S3. Sequence information for species used in the mock community. Accession numbers are provided for new sequences deposited from this study (bolded), and for those that have previously been deposited onto Genbank for the MiFish region (italics and starred). For those extracts not previously deposited, we list BLAST results for the MiFishU region (for fish) or for the control region (for cetaceans) of our newly generated sequences. Note that Ceratoscopelus townsendi, Physeter catodon and Ziphius cavirostris are omitted as they produced poor quality Sanger sequence data; and Hippoglossus stenolepis is omitted because we did not Sanger sequence that extract. Sequences belonging to the species marked with 'Zenodo' can be found in the accompanying datafiles to the manuscript deposited on Zenodo (see explanation above).

Species	Gene	Percent Identity of region on NCBI BLAST (if not previously deposited)	Accession Number
Carcharodon carcharias	12S		**PV221274**<br>*OQ846648.1**
Clupea pallasii	12S	100% to *C. pallasii*	**PV221278**
Diogenichthys atlanticus	12S	100% to *D. atlanticus*	**PV221275**
Engraulis mordax	12S		**PV221277**<br>*OQ846057.1**
Leuroglossus stilbius	12S	99.41% to *L. stilbius* and *L. schmidti*	**PV221281**
Merluccius productus	12S	100% to *M. productus* and *M. angustimanus*	**PV221276**
Oncorhynchus nerka	12S	100% to *O. nerka*	**PV221283**
Oncorhynchus tshawytscha	12S	100% to *O. tshawytscha*	**PV221284**
Sardinops sagax	12S		**PV221279**<br>*OQ846122.1**
Thaleichthys pacificus	12S	100% to *T. pacificus*	**PV221282**
Trachurus symmetricus	12S		**PV221280**<br>*OQ846073.1**
Balaenoptera acutorostrata	CR	98.41% to *B. acutorostrata*	Zenodo
Balaenoptera musculus	CR	100% to *B. musculus*	**PV268269**
Balaenoptera physalus	CR	98.18% to *B. physalus*	**PV268270**
Delphinus delphis	CR	99.79% to *D. delphis*	**PV268272**
Globicephala macrorhynchus	CR	100% to *G. macrorhynchus*	**PV268274**
Grampus griseus	CR	99.38% to *G. griseus*	**PV268273**
Megaptera novaeangliae	CR	99.79% to *M. novaeangliae*	Zenodo
Mesoplodon densirostris	CR	99.10% to *M. densirostris*	**PV268271**
Orcinus orca	CR	100% to *O. orca*	Zenodo
Peponocephala electra	CR	100% to *P. electra*	**PV268275**
Phocoena phocoena	CR	100% to *P. Phocoena*	Zenodo
Phocoenoides dalli	CR	100% to *P. dalli*	Zenodo

After confirming species identity for this subset of species used in the main paper analyses, we recalculated expected proportions for the subset community, based on both total genomic DNA (via Qubit) and template mtDNA concentrations (via Droplet Digital PCR [ddPCR], see below), and these new proportions are used in the main paper (Figure S2).

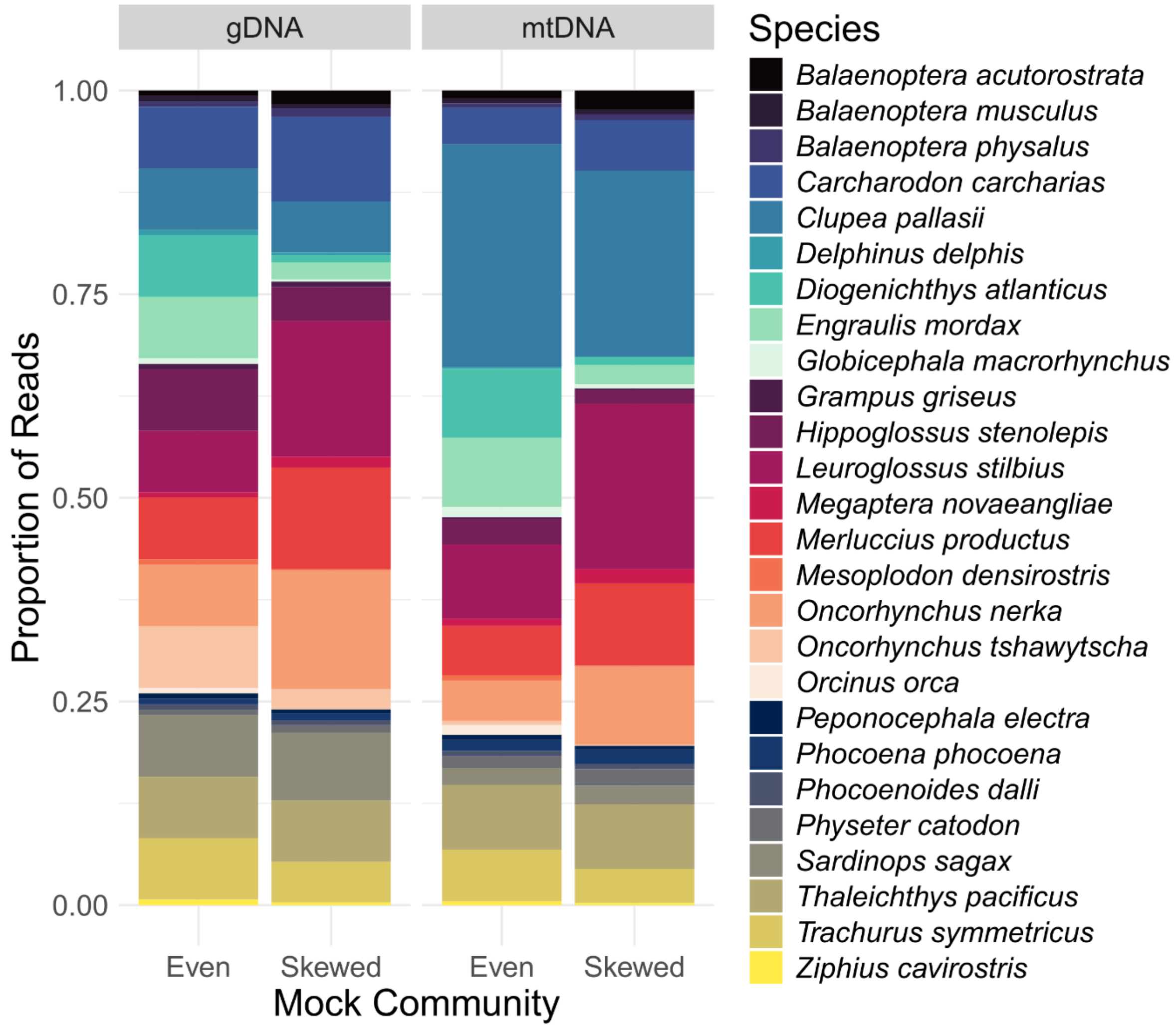

Figure S2. Expected proportions of the mock community subset containing only fishes and cetaceans used in the main paper for both the even and skewed communities.

S2. DNA Metabarcoding of the Mock Community

S2.1. DNA Metabarcoding Methods

S2.1.1. Treatments of the Mock Community

In total, we sequenced the even mock community in triplicate for 24 treatments of different primers, Taq polymerases and, for one marker (MiFishU), different PCR conditions, as described in the main paper and as shown in Figure S3. We also amplified the skewed

community with MarVer1 and MiFishU, with the NEB Phusion HiFi Taq and the addition of BSA (totalling two treatments).

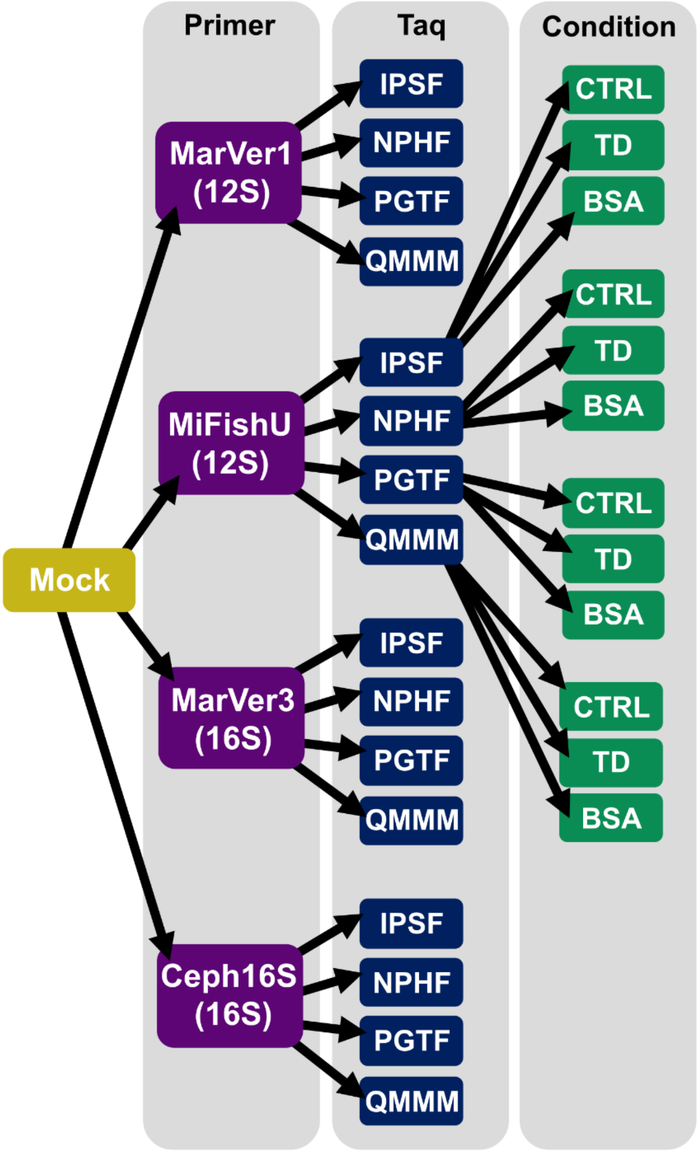

Figure S3. The treatments used to amplify the even mock community, where the Taqs are: Promega Go Taq Flexi (PGTF), Qiagen Multiplex Master Mix (QMMM), Invitrogen Platinum Superfi (IPSF), and NEB Phusion HiFi (NPHF). PCR Conditions are either with no BSA and normal cycling (i.e., the control, CTRL), with BSA added and normal cycling conditions (+BSA), or with no BSA and touchdown cycling (TD-PCR). All treatments were sequenced in triplicate.

S2.1.2. Amplification of the Mock Community

For amplification of the mock community, we chose 60°C as the annealing temperature because, after performing a gradient PCR from 48°C to 60°C on all marker-Taq combinations and visualizing the product on an agarose gel, 60°C was the temperature that produced a single strong product band with no nonspecific amplification for all marker-Taq combinations. Cycling conditions and recipes are listed in Table S4 and Table S5.

Table S4. Cycling conditions for all Taq polymerases used in this study.

	Promega GoTaq Flexi	Qiagen Multiplex Master Mix	NEB Phusion HiFi	Invitrogen Platinum Superfi
Initial denaturation	95°C for 2 min	95°C for 15 min	98°C for 30 sec	98°C for 30 sec
35 cycles of:<br>Denaturation<br>Annealing<br>Extension	95°C for 30 sec<br>60°C for 30 sec<br>72°C for 45 sec	94°C for 30 sec<br>60°C for 90 sec<br>72°C for 90 sec	98°C for 10 sec<br>60°C for 30 sec<br>72°C for 30 sec	98°C for 10 sec<br>60°C for 90 sec<br>72°C for 45 sec
Final extension	72°C for 5 min	72°C for 10 min	72°C for 10 min	72°C for 5 min

Table S5. Recipe conditions for all Taq polymerases used in this study. Bovine serum albumin (BSA) is in italics because some treatments of the mock community were run both with and without BSA.

Promega GoTaq Flexi		
Reagent	Volume for 20 µL rxn	Final concentration
GoTaq Flexi Buffer (5X)	4 µL	1X
Forward primer (10 µM)	0.4 µL	0.2 µM
Reverse primer (10 µM)	0.4 µL	0.2 µM
PCR nucleotide mix (10 nM each)	0.4 µL	0.2 nM each
GoTaq DNA Polymerase (5u/µL)	0.2 µL	1.25 u
MgCl2 (25 mM)	2.4 µL	3 mM
BSA (10 µg/µL)	*0.2 µL*	*0.1 µg/µL*
Nuclease-free water	10 µL	
Qiagen Multiplex Master Mix		
Qiagen Multiplex PCR Master Mix (2X)	10 µL	1X
Forward primer (10 µM)	0.4 µL	0.2 µM
Reverse primer (10 µM)	0.4 µL	0.2 µM
Q-Solution (5X)	4 µL	1X
BSA (10 µg/µL)	*1 µL*	*0.5 µg/µL*
Nuclease-free water	2.2 µL	
Template DNA	2 µL	
NEB Phusion HiFi		
Phusion Master Mix (2X)	10 µL	1X
Forward primer (10 µM)	1 µL	0.5 µM
Reverse primer (10 µM)	1 µL	0.5 µM
DMSO (100%)	0.6 µL	3%
BSA (10 ug/µL)	*1 µL*	*0.5 µg/µL*
Nuclease-free water	4.4 µL	
Template DNA	2 µL	
Invitrogen Platinum Superfi		
SuperFi II Master Mix (2X)	10 µL	1X
Forward primer (10 µM)	0.4 µL	0.2 µM
Reverse primer (10 µM)	0.4 µL	0.2 µM
BSA (10 µg/µL)	*1 µL*	*0.5 µg/µL*
Nuclease-free water	6.2 µL	
Template DNA	2 µL	

S2.1.3. Library Preparation and Sequencing

After the first PCR (PCR1), we bead cleaned the product using Ampure XP Beads (Beckman Coulter; 1x beads with two 80% ethanol washes before elution) to remove primer dimer. Before indexing, we visualized the product on a gel, quantified the product with Qubit Fluorometer and added roughly equal amounts (in ng) of input PCR1 product to the indexing reaction. We then indexed the product by attaching a unique 10 bp tag with P5 or P7 MiSeq flow cell adapter via the Nextera overhang using the IDT for Illumina DNA/RNA UD Indexes, following the recipe (per 25 µL rxn): 12.5 µL KAPA Hifi HotStart ReadyMix, 1.25 µL of the unique IDT for Illumina DNA/RNA UD Index (one per sample), and up to 11.25 of the bead cleaned PCR1 product (volume based on amount added from normalization, with the rest of 11.25 µL volume replaced with nuclease-free water). We used the following cycling conditions for the indexing reaction: initial denaturation of 95°C for 5 min; followed by 8 cycles of denaturation (98°C for 20 sec), annealing (56°C for 30 sec), extension (72°C for 1 min); and ending with a final extension of 72°C for 5 min. After the index reaction (PCR2), we bead cleaned the product again with Ampure XP Beads (0.8x beads with two 80% ethanol washes before elution). We quantified the final bead cleaned PCR2 product using Qubit Fluorometer using the ds High Sensitivity Assay Kit and then normalized all samples so they were equimolar in the final library pool.

We sequenced libraries on a MiSeq System (Illumina) using a v3 600 cycle kit. We ran samples across three different final pooled libraries that were run on three different sequencing runs, each containing ~100 samples. The first library contained all mock community samples. The second and third libraries contained approximately 20 mock community samples with the remaining samples belonging to other projects. Loading concentrations and percent of PhiX added for each run, as well as summaries of run properties, are in Table S6. Between runs, we performed template line washes and we used different sets of indexes on back to back runs to minimize cross run carryover of indexes.

Table S6. Library loading information and run summaries for all three MiSeq runs.

Library	Loading Concentration	Percent PhiX	Cluster Density	Percent Clusters Passing Filter	Percent PhiX Aligned
1	8	10%	895	93.5%	5.85%
2	8	15%	1029	88.7%	8.22%
3	6	20%	757	92.5%	13.09%

S2.1.4. Sequence Curation

We curated some ASVs based on knowledge of what we put into the mock community. If an ASV BLASTed equally to something we put into the mock and a closely related species, we assigned that ASV to the species in the mock community. This occurred for some cetaceans, particularly for delphinids belonging to the *Stenella-Tursiops-Delphinus* complex, which are notoriously difficult to resolve (Dizon, 2000). When an ASV matched a cetacean species in the mock community as well as another cetacean species not in the mock community (e.g., matched *Delphinus delphis* [in the mock] and *Tursiops truncatus* [not in mock]), we assigned the ASV to that cetacean species that was put into the mock community (e.g., to *Delphinus delphis*). There were some instances where an ASV matched multiple cetacean species included in the mock community (particularly for *Delphinus delphis, Orcinus orca, Globicephala macrorhynchus* and *Peponocephala electra*), and this ASV was left at Class level Mammalia. None of the minor ambiguities in assigning taxonomy to ASVs from the mock community would substantially affect the results we report.

S2.2. DNA Metabarcoding Results

S2.2.1. Bioinformatic Summary

Information for how many reads were retained after filtering, denoising, merging, and removing chimeras for all samples (samples of the even or skewed community in triplicate or more, totalling 100 samples) can be found in the Table S7. In total, we obtained 17,700,357 reads across all samples (read depth across samples for each marker can be found in Table S8).

Table S7. dada2 filter and merging statistics for all treatments of the mock community. Naming convention as "marker.Taq.treatment(if applicable).replicate". For markers, MV1 = MarVer1 (12S), MV3 = MarVer3 (16S), MFU = MiFishU (12S) and C16 = Ceph16S (16S). For Taqs, IPSF = Invitrogen Platinum SuperFi, NPHF = NEB Phusion HiFi, PGTF = Promega GoTaq Flexi and QMMM = Qiagen Multiplex Master Mix. Sample denoted if the reaction contained BSA or was cycled using touchdown cycling (TD).

Sample	input	filtered	denoisF	denoisR	merged	nonchim
C16.IPSF.1	292288	283209	283048	283130	282668	271247
C16.IPSF.2	460942	446273	446091	446122	445538	429160
C16.IPSF.3	245578	236918	236724	236802	235428	227225
C16.NPHF.1	256743	244352	243902	244041	241242	211973
C16.NPHF.2	220605	210275	209901	209832	207590	182286
C16.NPHF.3	256231	244141	243773	243840	241296	212265
C16.PGTF.BSA.1	195692	187656	187383	187412	186267	181520
C16.PGTF.BSA.2	169951	163670	163353	163441	162020	156578
C16.PGTF.BSA.3	237110	228240	227905	228134	226411	219208
C16.QMMM.1	203410	197532	197390	197411	196318	187188
C16.QMMM.2	157572	153092	152985	152988	152117	144881
C16.QMMM.3	316190	306635	306531	306370	304179	292917
MFU.IPSF.1	132358	127783	127649	127661	127276	121474
MFU.IPSF.2	128993	123724	123549	123582	123092	118757
MFU.IPSF.3	126354	121342	121170	121252	120788	117189
MFU.IPSF.BSA.1	144877	126608	126483	126396	125710	113132
MFU.IPSF.BSA.2	164128	134121	133932	133969	133211	120870
MFU.IPSF.BSA.3	140122	119514	119257	119387	118486	105225
MFU.IPSF.TD.1	132045	114817	114563	114596	113436	95582
MFU.IPSF.TD.2	110790	95316	95146	95155	94124	79828
MFU.IPSF.TD.3	92042	78713	78401	78551	77556	65439
MFU.mock.skewed.BSA.1	92269	86929	86765	86796	81840	71641
MFU.mock.skewed.BSA.2	351896	331914	331503	331582	313730	270939
MFU.mock.skewed.BSA.3	291115	271914	271524	271571	256789	222886
MFU.mock.skewed.BSA.TD.1	579337	539240	538573	538780	531357	434447
MFU.mock.skewed.BSA.TD.2	625430	596481	595735	596044	588466	480564
MFU.mock.skewed.BSA.TD.3	649207	614581	613589	613968	605637	492872
MFU.NPHF.1	165919	160327	159834	160112	158688	135111
MFU.NPHF.2	200071	193033	192627	192833	191182	161521
MFU.NPHF.3	202472	195768	195428	195530	194079	165250
MFU.NPHF.BSA.1	219236	205573	205314	205283	192676	170187
MFU.NPHF.BSA.2	191496	181170	180797	180932	169399	149597
MFU.NPHF.BSA.3	141486	126779	126573	126574	117529	101558
MFU.NPHF.TD.1	496365	471755	470883	471299	465490	388143

MFU.NPHF.TD.2	781485	738320	737616	737715	730228	609530
MFU.NPHF.TD.3	445659	424896	424343	424530	419934	354850
MFU.NPHF.TD.4	110109	92724	92487	92559	91591	75238
MFU.NPHF.TD.5	137298	117536	117318	117347	116321	95495
MFU.NPHF.TD.6	184387	158309	157953	158107	156546	126659
MFU.NTC	276	2	2	2	0	0
MFU.PGTF.1	124471	100009	99849	99774	99275	93677
MFU.PGTF.2	145417	126295	125988	126123	125427	118217
MFU.PGTF.3	139843	119718	119463	119566	118853	110962
MFU.PGTF.BSA.1	194726	187886	187505	187541	186329	161117
MFU.PGTF.BSA.1	163515	138867	138634	138630	137818	128599
MFU.PGTF.BSA.2	118394	95569	95383	95464	94822	89362
MFU.PGTF.BSA.2	183346	176305	175749	176122	174712	152188
MFU.PGTF.BSA.3	222759	214521	213698	214230	212254	183867
MFU.PGTF.BSA.3	107650	92325	92057	92203	91530	85158
MFU.PGTF.TD.1	130309	110332	110140	110149	109455	101647
MFU.PGTF.TD.2	148171	126236	125936	125975	124987	115424
MFU.PGTF.TD.3	95617	81574	81396	81309	80623	74097
MFU.QMMM.1	175689	166264	166102	166119	165343	154275
MFU.QMMM.2	158344	151033	150870	150830	150130	139018
MFU.QMMM.3	123063	116693	116406	116433	115765	108608
MFU.QMMM.BSA.1	551933	520482	519667	519993	513826	469769
MFU.QMMM.BSA.2	522718	494635	493916	493914	488611	445375
MFU.QMMM.BSA.3	598672	567413	566982	567105	560860	503919
MFU.QMMM.BSA.4	137546	120818	120613	120608	119938	112675
MFU.QMMM.BSA.5	140594	118573	118292	118438	117742	109643
MFU.QMMM.BSA.6	154725	130838	130678	130638	129859	120495
MFU.QMMM.TD.1	183033	177388	176820	177176	175770	159824
MFU.QMMM.TD.2	142891	136256	136038	136096	135221	123524
MFU.QMMM.TD.3	139868	133968	133829	133743	132796	120657
MV1.IPSF.1	199746	192187	191814	192033	190949	184944
MV1.IPSF.2	179021	173247	172998	173092	172174	166580
MV1.IPSF.3	113597	107710	107550	107597	107001	104236
MV1.mock.skewed.BSA.1	263376	247234	246560	246870	233549	199201
MV1.mock.skewed.BSA.2	264051	247191	246482	246796	233633	198241
MV1.mock.skewed.BSA.3	196523	185581	185048	185323	175581	149140
MV1.NPHF.1	219919	212340	211829	212102	209644	178783
MV1.NPHF.2	212988	206230	205711	205846	202875	170172
MV1.NPHF.3	112956	107819	107432	107665	105847	89663
MV1.NPHF.BSA.1	252917	235798	235025	235301	224185	194528
MV1.NPHF.BSA.2	209824	194402	193882	193886	184650	160572
MV1.NPHF.BSA.3	222619	209347	208762	208990	198426	168274
MV1.PGTF.BSA.1	242044	227915	227488	227503	224943	210354
MV1.PGTF.BSA.2	206001	197932	197311	197581	195162	181750
MV1.PGTF.BSA.3	168450	162844	162444	162499	160558	150889
MV1.PGTF.BSA.TD.1	214522	205966	205513	205666	203062	182933
MV1.PGTF.BSA.TD.2	134295	128160	127870	127926	126353	114925
MV1.PGTF.BSA.TD.3	188556	181922	181603	181544	179665	162636
MV1.QMMM.1	181054	173574	173251	173397	172213	164401
MV1.QMMM.2	222563	214131	213787	213940	212415	202451
MV1.QMMM.3	149290	143422	143014	143101	141783	136033

MV3.IPSF.1	291821	275740	275422	275473	274079	259306
MV3.IPSF.2	46992	44763	44620	44697	44303	42263
MV3.IPSF.3	41508	39356	39156	39244	38788	37358
MV3.NPHF.1	384748	365146	364377	364572	357516	283278
MV3.NPHF.2	92871	88256	87818	88110	85921	69570
MV3.NPHF.3	119333	109293	108868	108899	106358	88043
MV3.PGTF.BSA.1	91920	87627	87219	87438	86015	76039
MV3.PGTF.BSA.2	207161	196590	196121	196298	193974	170308
MV3.PGTF.BSA.3	102966	98491	98171	98317	96957	84710
MV3.PGTF.BSA.TD.1	220741	208479	208131	208191	205650	177662
MV3.PGTF.BSA.TD.2	121273	114765	114457	114565	112927	99239
MV3.PGTF.BSA.TD.3	161235	152493	152089	152259	150240	130167
MV3.QMMM.1	332858	313724	313328	313503	311794	286273
MV3.QMMM.2	151583	144424	144008	144261	143064	129960
MV3.QMMM.3	179764	170920	170487	170593	169261	152946

The samples amplified by Ceph16S had the highest mean read depth, while MarVer1, MiFishU and MarVer3 samples contained similar mean read depths. Over 99% of reads went to the species that we originally put into the larger mock community (e.g., containing cetaceans, fish, cephalopods and krill). When considering only the subset of the mock community containing fishes and cetaceans, for Ceph16S, only 30% of reads went to fish and cetaceans, where the rest went largely to cephalopods. Instead, MarVer1, MarVer3 and MiFishU samples contained no reads to cephalopods or krill, and nearly all reads were assigned to species in the subset of the mock community. We found that a low percentage of reads went to species that we did not put into our mock community (see below).

Table S8. Read depths for samples for each marker, with the total number of samples run given below each marker. Note the NTC has been excluded for these stats as no reads passed filter. The min and max are the minimum and maximum reads assigned to a sample for that primer set, and the mean and medians are given for all samples run for each primer set. N_{ASVs} refers to the number of ASVs retained after dada2 and the percent in the mock is the percentage of the reads that assigned to a species in the entire mock community containing fishes, cetaceans, cephalopods and krill. The percent outside the expected was the percent of ASVs assigned to a species that was not put into the mock community or to an unassigned ASV. Note for Ceph16S, one of the most abundant ASV was not able to be assigned to species level likely due to missing reference sequences (as it only had 92% percent identity in blast to a congener of the mock); updated percentages are given in parentheses if we assume that that top cephalopod sequence belonged to a species in the mock.

Marker	Total	Min	Max	Mean	Median	N_{ASVs}	% Reads in Mock	% Reads Outside Expected
Ceph16S (N = 12)	2716448	144881	429160	226370.6 ± 76886.0	212119	86	70.88 (99.93)	29.12 (0.07)
MiFishU (N = 51)	9426081	65439	609530	184825.1 ± 138791.3	121474	155	99.96	0.04
MarVer1 (N = 21)	3470706	89663	210354	165271.7 ± 32423.12	168274	137	99.77	0.23
MarVer3 (N = 15)	2087122	37358	286273	139141.5 ± 82551.5	129960	108	99.87	0.13

S2.2.2. Technical Replication

We performed triplicate technical PCR replicates to assess variability that may be attributable to pipette error. Triplicate replicates showed that the metabarcoding output for a given treatment was reproducible for each Marker-Taq combination (Figure S4).

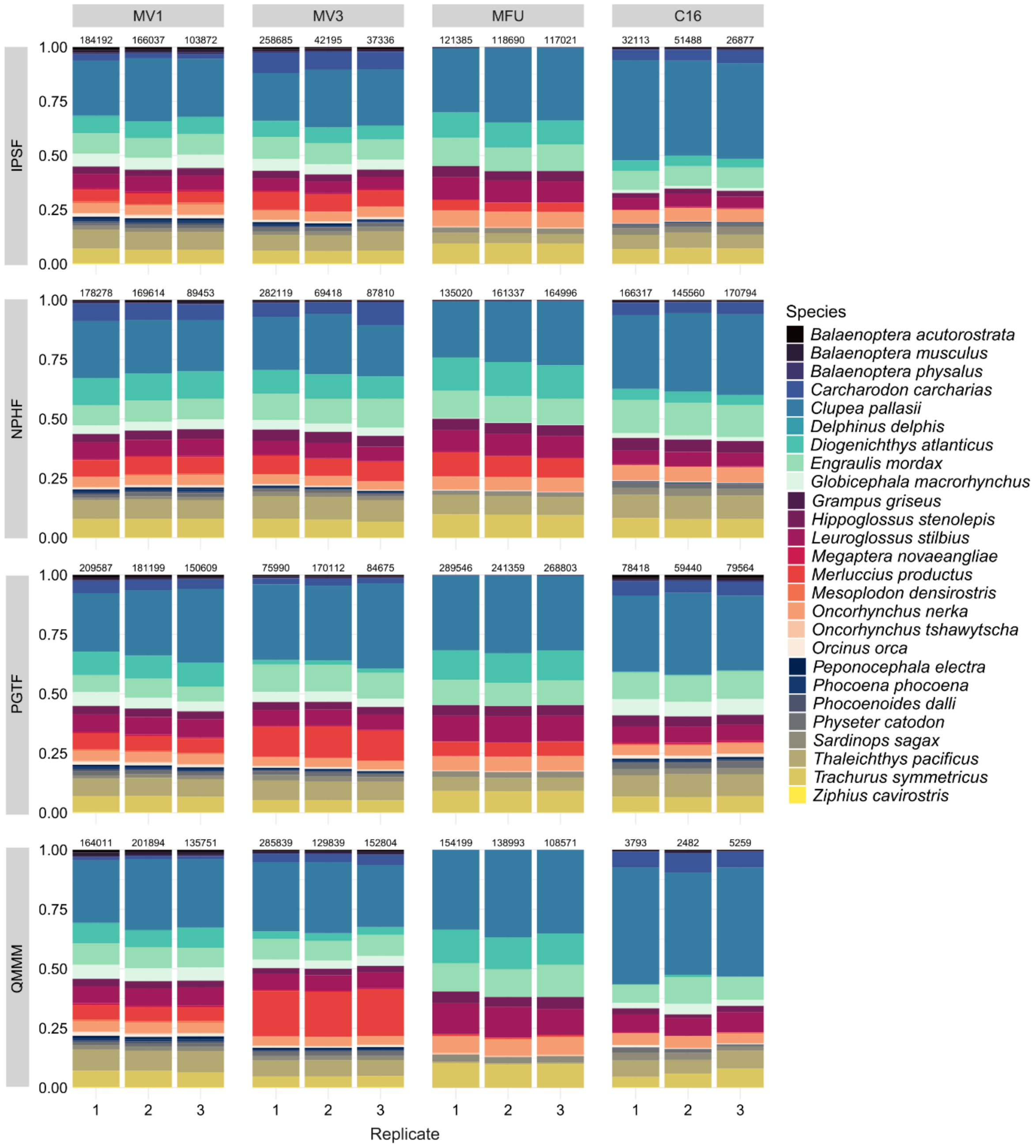

Figure S4. Technical PCR replicates for the mock community for all Taq polymerases for all markers run. Markers are abbreviated as the following: MV1 = MarVer1 (12S), MV3 = MarVer3 (16S), MFU = MiFishU (12S), and C16 = Ceph16S (16S). Taqs are abbreviated as the following: IPSF = Invitrogen Platinum SuperFi; NPHF = NEB Phusion HiFi; PGTF = Promega GoTaq Flexi; QMMM = Qiagen Multiplex Master Mix. Read depth for each sample is listed above each replicate's bar. Plots contain species in the subset mock community used in the main paper.

S2.2.3. Off Targets: Assignment to Species Outside of the Mock Community

For Ceph16S, one of most abundant ASVs was unable to be assigned to species-level and had a percent identity in blast of 92% to *Histioteuthis bonnellii*. This species is a conger of the mock community member *Histioteuthis heteropsis*, which at the time of writing this paper, does not have a 16S reference sequence publicly available. It is therefore likely that this ASV belongs to that species. We therefore remove it from the off target analysis we summarize here: of the total 86 Ceph16S ASVs, 32 were either unassigned or assigned to species outside of the mock community (which made up 0.07% of the total Ceph16S reads), which ranged from containing 2 to 239 reads (mean = 25.7 ± 43.9; median = 11). For off target ASVs that got assigned to species level, these included: *Lampadena urophaos*, *Lepidoteuthis grimaldii*, *Lipolagus ochotensis*, *Nannobrachium regale* and *Rathbunaster californicus.*

For MiFishU, 91 of 155 ASVs were either unassigned or assigned to species outside of the mock community (which made up 0.04% of total reads for MiFishU), ranging from 2 to 167 reads (mean = 10.8 ± 15.9, median = 6). Assignments to species not contained in the mock community included: *Anoplopoma fimbria*, *Centrobranchus nigroocellatus*, *Diaphus anderseni*, *Diaphus theta*, *Dicamptodon tenebrosus*, *Diplospinus multistriatus*, *Elops affinis*, *Gasterosteus aculeatus*, *Ichthyococcus irregularis*, *Icichthys lockingtoni*, *Lampadena urophaos*, *Lampanyctus tenuiformis*, *Lampetra sp.* CBM:ZF:17561, *Lipolagus ochotensis*, *Lycodes cortezianus*, *Lyconema barbatum*, *Melamphaes lugubris*, *Microstomus pacificus*, *Nannobrachium regale*, *Oncorhynchus clarkii*, *Ophiodon elongatus*, *Perca flavescens*, *Photonectes margarita*, *Plectroglyphidodon aureus*, *Scopelogadus bispinosus*, *Seriola lalandi*, *Stomias atriventer*, *Symbolophorus californiensis*, *Triphoturus mexicanus*, *Triphoturus nigrescens*, *Turdus migratorius* and *Xeneretmus latifrons*

For MarVer1, 87 ASVs of the 137 total were assigned to species outside of the mock community or unassigned (which made up 0.23 % of total reads for MarVer1). These ASVs ranged from having 2 to 205 reads (mean = 23.1 ± 27.9, median = 14). The off target ASVs that were assigned to species level were as follows: *Ameiurus nebulosus*, *Anoplopoma fimbria*, *Diaphus theta*, *Dicamptodon tenebrosus*, *Homo sapiens*, *Icichthys lockingtoni*, *Lampetra sp.* CBM:ZF:17561, *Lipolagus ochotensis*, *Oncorhynchus clarkii*, *Oncorhynchus kisutch*, *Ophiodon elongatus*, *Procyon lotor*, *Seriola lalandi*, *Stomias atriventer*, *Triphoturus mexicanus* and*Turdus migratorius*

For MarVer3, 55 of 108 ASVs were either unassigned to assigned to species not in the mock community (which made up 0.13% of total reads for MarVer3), and these ranged from 2 to 285 reads (mean = 19.0 ± 27.3, median = 11). The ASVs assigned to species included: *Anoplopoma fimbria*, *Gonatus fabricii*, *Lipolagus ochotensis*, *Nannobrachium regale*, *Nezumia stelgidolepis*, *Ophiodon elongatus* and *Sebastolobus altivelis*.

We suspect the low read assignments are due to contamination of the tissue or extract and/or due to very small amount of carryover of reads from previous runs (as for the case with MiFishU, where some freshwater species appeared [with few reads] that contained the same indexes from a previous run containing freshwater species). We do not believe that these low levels of contamination (where off targets comprised of 0.23% or less of reads for a given marker) significantly altered the analysis and findings we present in the main paper.

S3. Quantifying Mitochondrial DNA Template Concentration of Mock Community Species Extracts with ddPCR

S3.1. ddPCR Methods

In order to get template concentration (copies/µL) rather than genomic concentration (ng/µL) for each species in the mock community, we quantified all extracts using droplet digital PCR (ddPCR). We first determined the input concentration (0.05 ng/µL) for all extracts by testing a subset of the extracts at 0.5 ng/µL, 0.05 ng/µL and 0.005 ng/µL. We found that 0.05 ng/µL gave us an appropriate ratio of positive to negative droplets and chose this as our starting concentration. We normalized all of our extracts to either 0.25 ng/µL or 0.5 ng/µL (depending on how much extract we had left) and then quantified them using the ds High Sensitivity Assay Kit on Qubit Fluorometer. Concentrations were within 0.1 ng/µL of the expected concentration. We then diluted these to 0.05 ng/µL as input for ddPCR (but could not quantify as 0.05 ng/µL is out of the range of the Qubit HS assay). The input concentration was assumed to be the quantification from the normalized Qubit (e.g., approximately 0.25 or 0.5) divided by the dilution factor. The final concentrations from ddPCR were then corrected for by dividing by the input concentration – giving a final concentration of mitochondrial copies per ng of genomic DNA input.

We performed ddPCR in 22 µL reactions, with the following final concentrations: 1X QX200 ddPCR EvaGreen Supermix, 0.2 µM forward primer, 0.2 µM reverse primer, with 2 µL of DNA [at 0.05 ng/µL] and the remaining volume to 22 µL with nuclease-free water. We performed this reaction for each primer set analysed in the main paper (MarVer1, MiFishU, MarVer3, Ceph16S). We amplified droplets using the following cycling conditions: enzyme activation at 95°C for 5 min, followed by 40 cycles of denaturation (95°C for 30 sec) and anneal/extension (60°C for 1 min), followed by signal stabilization (4°C for 5 min, 90°C for 5 min).

S3.2. ddPCR Results

In general, the ddPCR concentration of a species for a marker that contained mismatches to the primer binding site was underestimated compared to a primer set that contained perfect matches (Figure 2 in main paper). While ddPCR generally does not depend on efficiency (i.e., it is endpoint PCR), we found evidence that the number of positive droplets decreased if the species had a lower affinity to bind to the primer. We did not get reliable ddPCR concentrations for *Ceratoscopelus townsendi* for any of the four markers. We also failed to get a concentration estimate from MiFishU for *Phocoena phocoena* due to error using the Droplet Digital PCR instrument and we could not rerun the sample due to extract limitations.

We then calculated the proportion of mitochondrial template DNA to total genomic DNA (ng mtDNA: ng gDNA) for each species in the following manner. We first converted the concentration in copies/µL for each species given from MarVer1 (which contained a perfect match to the primer binding sites of all species in the mock, and so we assume is an estimate for concentration of mtDNA) to ng/µL by multiplying the copies/µL by the length of fragment (163 bp), the average mass of 1bp of dsDNA (660 g/mol) and a conversion factor of 1 x 10^9 ng/g, and then divided this by Avogadro's number (6.0221 x 10^{23} molecules/mole). We then divided this by the Qubit input into the ddPCR reaction (which was approximately 0.05 ng/ul for each species).

We found that the expected proportion of species changed in the mixture after recalculating the species proportion based on mtDNA concentration rather than total gDNA (via Qubit). The percentage of mtDNA of gDNA proportions in the mock community revealed no pattern (Figure S5). *Clupea pallasii* contained the highest percentage based on mtDNA compared to the other species, and *Oncorhynchus tshawytscha* the lowest.

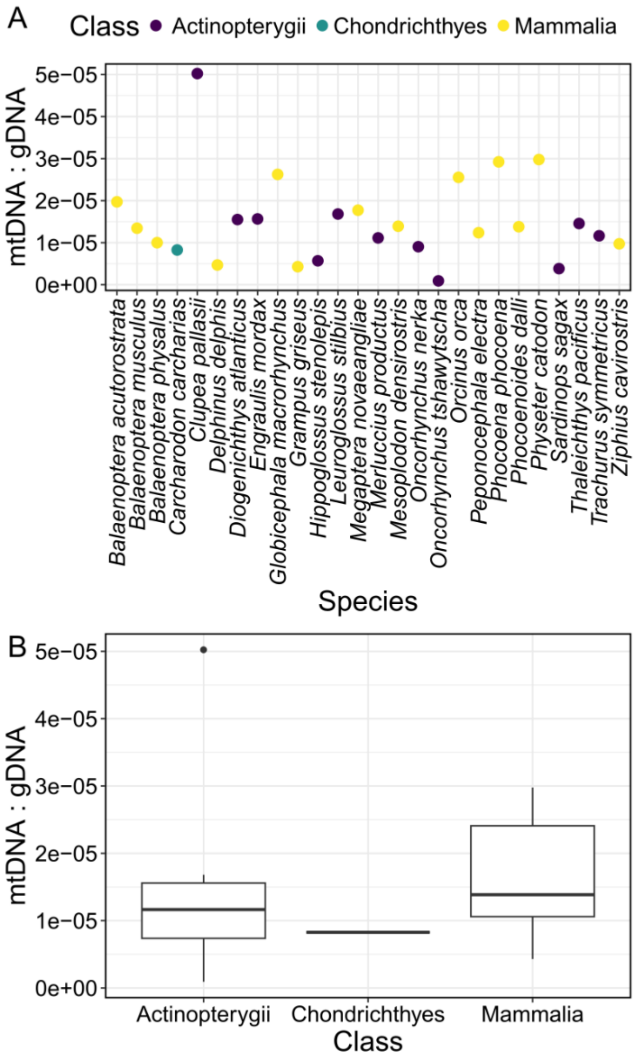

Figure S5. Ratio of mtDNA (in ng/µL, converted from MarVer1 copies/µL via ddPCR) amplified from the total gDNA (in ng/µL, via Qubit Fluorometry) inputted into the ddPCR reaction for each species in the mock community, by species (A) and by class (B). Plots contain species in the subset mock community used in the main paper.

S4. Characterizing Potential Contributors to Amplification Efficiency: Mismatches, GC Content and Fragment Length for Mock Community Species

S4.1. Mismatches between Primer-Template Pairs

To determine the number of mismatches between each primer-template pair for each species and marker, we performed a virtual PCR (via the *insect* package in R, Wilkinson et al., 2018) to identify the primer regions within templates, and then aligned all resulting amplicons in Geneious Prime. Template sequences were derived from Sanger sequences we generated of the longer 12S fragment that nests MarVer1 and MiFishU (see 1.2. Species Confirmation of the Mock Community), or were estimates based upon GenBank accessions for the target taxa (16S). We counted mismatches as the absolute number of differences between primer and consensus template sequences. When there were multiple reference sequences and no unambiguous consensus at the primer binding site, we chose the primer binding site of the sequence that matched the ASV that we obtained from the mock community for that species. When the mock community sequence was the same for multiple sequences in the alignment and there was still no consensus at the primer binding site (a rare instance), we used the primer binding site sequence that was the most common (i.e., had the most reference sequences on GenBank). The total number of mismatches between primer-template pairs for each species for each marker can be found in Table S9.

S4.2. GC Content and Fragment Length

Using the amplicons generated for the alignments (without the primer binding sites), we calculated GC content for each amplicon for all species-primer set combinations using IDT OligoAnalyzer, where GC content is calculated as the molar percentage of guanine and cytosine bases in a sequence. We also calculated the fragment length for each amplicon.

Table S9. Amplicon information for each species in the subset mock community. GC content and fragment length do not contain the forward and reverser primer binding sites. Total number of mismatches refers to the sum of mismatches in the forward and reverse binding sites for each species in the mock community.

Species	Class	Marker	GC Content (%)	Length (bp)
Ceratoscopelus townsendi	Actinopteri	Ceph16S	53.2	217
Clupea pallasii	Actinopteri	Ceph16S	50.7	217
Diogenichthys atlanticus	Actinopteri	Ceph16S	52.9	206
Engraulis mordax	Actinopteri	Ceph16S	45	218
Hippoglossus stenolepis	Actinopteri	Ceph16S	50.6	231
Leuroglossus stilbius	Actinopteri	Ceph16S	49.5	218
Merluccius productus	Actinopteri	Ceph16S	42.6	216
Oncorhynchus nerka	Actinopteri	Ceph16S	52.5	219
Oncorhynchus tshawytscha	Actinopteri	Ceph16S	52.7	220
Sardinops sagax	Actinopteri	Ceph16S	52.1	213
Thaleichthys pacificus	Actinopteri	Ceph16S	49.3	217
Trachurus symmetricus	Actinopteri	Ceph16S	47.1	239
Carcharodon carcharias	Chondrichthyes	Ceph16S	31.4	226
Balaenoptera acutorostrata	Mammalia	Ceph16S	39.3	191

Balaenoptera musculus	Mammalia	Ceph16S	38.7	191
Balaenoptera physalus	Mammalia	Ceph16S	40.3	191
Delphinus delphis	Mammalia	Ceph16S	36.1	191
Globicephala macrorhynchus	Mammalia	Ceph16S	36.1	191
Grampus griseus	Mammalia	Ceph16S	36.1	191
Megaptera novaeangliae	Mammalia	Ceph16S	39.6	192
Mesoplodon densirostris	Mammalia	Ceph16S	34	194
Orcinus orca	Mammalia	Ceph16S	37.2	191
Peponocephala electra	Mammalia	Ceph16S	36.6	191
Phocoena phocoena	Mammalia	Ceph16S	36.3	193
Phocoenoides dalli	Mammalia	Ceph16S	36.6	194
Physeter catodon	Mammalia	Ceph16S	42.7	192
Ziphius cavirostris	Mammalia	Ceph16S	39.1	192
Ceratoscopelus townsendi	Actinopteri	MiFishU	54.1	172
Clupea pallasii	Actinopteri	MiFishU	54.1	172
Diogenichthys atlanticus	Actinopteri	MiFishU	52.1	169
Engraulis mordax	Actinopteri	MiFishU	46.8	173
Hippoglossus stenolepis	Actinopteri	MiFishU	45.6	169
Leuroglossus stilbius	Actinopteri	MiFishU	52.7	169
Merluccius productus	Actinopteri	MiFishU	44.4	169
Oncorhynchus nerka	Actinopteri	MiFishU	49.1	169
Oncorhynchus tshawytscha	Actinopteri	MiFishU	49.4	170
Sardinops sagax	Actinopteri	MiFishU	48	173
Thaleichthys pacificus	Actinopteri	MiFishU	47	168
Trachurus symmetricus	Actinopteri	MiFishU	46.5	170
Carcharodon carcharias	Chondrichthyes	MiFishU	39	182
Balaenoptera acutorostrata	Mammalia	MiFishU	36.3	171
Balaenoptera musculus	Mammalia	MiFishU	35.3	170
Balaenoptera physalus	Mammalia	MiFishU	37.6	170
Delphinus delphis	Mammalia	MiFishU	34.5	171
Globicephala macrorhynchus	Mammalia	MiFishU	33.3	171
Grampus griseus	Mammalia	MiFishU	35.7	171
Megaptera novaeangliae	Mammalia	MiFishU	38.2	170
Mesoplodon densirostris	Mammalia	MiFishU	32.4	170
Orcinus orca	Mammalia	MiFishU	33.3	171
Peponocephala electra	Mammalia	MiFishU	33.9	171
Phocoena phocoena	Mammalia	MiFishU	40.6	170
Phocoenoides dalli	Mammalia	MiFishU	41.2	171
Physeter catodon	Mammalia	MiFishU	39.1	169
Ziphius cavirostris	Mammalia	MiFishU	32.4	170
Ceratoscopelus townsendi	Actinopteri	MarVer1	52.7	165
Clupea pallasii	Actinopteri	MarVer1	52.7	165
Diogenichthys atlanticus	Actinopteri	MarVer1	50.6	162
Engraulis mordax	Actinopteri	MarVer1	45.2	166
Hippoglossus stenolepis	Actinopteri	MarVer1	43.8	162
Leuroglossus stilbius	Actinopteri	MarVer1	51.2	162
Merluccius productus	Actinopteri	MarVer1	42.6	162
Oncorhynchus nerka	Actinopteri	MarVer1	47.5	162
Oncorhynchus tshawytscha	Actinopteri	MarVer1	47.9	163
Sardinops sagax	Actinopteri	MarVer1	46.4	166
Thaleichthys pacificus	Actinopteri	MarVer1	45.3	161

Trachurus symmetricus	Actinopteri	MarVer1	44.8	163
Carcharodon carcharias	Chondrichthyes	MarVer1	37.1	175
Balaenoptera acutorostrata	Mammalia	MarVer1	34.1	164
Balaenoptera musculus	Mammalia	MarVer1	33.1	163
Balaenoptera physalus	Mammalia	MarVer1	35.6	163
Delphinus delphis	Mammalia	MarVer1	32.3	164
Globicephala macrorhynchus	Mammalia	MarVer1	31.1	164
Grampus griseus	Mammalia	MarVer1	33.5	164
Megaptera novaeangliae	Mammalia	MarVer1	36.2	163
Mesoplodon densirostris	Mammalia	MarVer1	30.1	163
Orcinus orca	Mammalia	MarVer1	31.1	164
Peponocephala electra	Mammalia	MarVer1	31.7	164
Phocoena phocoena	Mammalia	MarVer1	38.7	163
Phocoenoides dalli	Mammalia	MarVer1	39.3	164
Physeter catodon	Mammalia	MarVer1	37	162
Ziphius cavirostris	Mammalia	MarVer1	30.1	163
Ceratoscopelus townsendi	Actinopteri	MarVer3	53.4	223
Clupea pallasii	Actinopteri	MarVer3	50.7	223
Diogenichthys atlanticus	Actinopteri	MarVer3	52.4	212
Engraulis mordax	Actinopteri	MarVer3	45.1	224
Hippoglossus stenolepis	Actinopteri	MarVer3	50.6	237
Leuroglossus stilbius	Actinopteri	MarVer3	49.6	224
Merluccius productus	Actinopteri	MarVer3	42.8	222
Oncorhynchus nerka	Actinopteri	MarVer3	52.4	225
Oncorhynchus tshawytscha	Actinopteri	MarVer3	52.7	226
Sardinops sagax	Actinopteri	MarVer3	52.1	219
Thaleichthys pacificus	Actinopteri	MarVer3	49.3	223
Trachurus symmetricus	Actinopteri	MarVer3	47.1	245
Carcharodon carcharias	Chondrichthyes	MarVer3	31.9	232
Balaenoptera acutorostrata	Mammalia	MarVer3	39.6	197
Balaenoptera musculus	Mammalia	MarVer3	39.1	197
Balaenoptera physalus	Mammalia	MarVer3	40.6	197
Delphinus delphis	Mammalia	MarVer3	36.5	197
Globicephala macrorhynchus	Mammalia	MarVer3	36.5	197
Grampus griseus	Mammalia	MarVer3	36.5	197
Megaptera novaeangliae	Mammalia	MarVer3	39.9	198
Mesoplodon densirostris	Mammalia	MarVer3	34.5	200
Orcinus orca	Mammalia	MarVer3	37.6	197
Peponocephala electra	Mammalia	MarVer3	37.1	197
Phocoena phocoena	Mammalia	MarVer3	36.7	199
Phocoenoides dalli	Mammalia	MarVer3	37	200
Physeter catodon	Mammalia	MarVer3	42.9	198
Ziphius cavirostris	Mammalia	MarVer3	39.4	198

S5. Measuring Observation Bias

S5.1. Modelling Amplification Efficiency for Species in the Even Mock Community Subset

S5.1.1. Model Summaries and Selection

We investigated the relationship between amplification efficiency of a species with predictors previously reported to be informative, including number of mismatches between the primer and species template, GC content, amplicon length and Taq polymerase used (Dabney & Meyer, 2011; Huber et al., 2009; Nichols et al., 2018; SantaLucia & Hicks, 2004; Stadhouders et al., 2010). We employed a linear regression model implemented in a Bayesian framework, using the 'stan_lm' function from the 'rstanarm package' (Goodrich et al., 2024) in R (R Core Team, 2023). We first measured amplification efficiency for each species and marker-Taq combination, using the quantitative metabarcoding model from Shelton et al. (2023), calibrated with the expected species proportions based upon mtDNA concentrations, as described in the main paper. The model was run separately for each marker-Taq and yielded an amplification efficiency for each species-marker-Taq combination (Figure S6). Because the amplification efficiency parameters are on a common scale within each marker – the estimated parameter is the log-ratio of each individual species relative to that of an arbitrary reference species (here, *Engraulis mordax*) – it is possible to combine information across loci and Taq polymerases to create a generalizable analysis. We combined the findings for all markers and then tested the following base model to first identify predictors that may be meaningful:

$$\alpha_{ijk} = \beta_1 \mathrm{mm}_{ij} + \beta_2 \mathrm{GC}_{ij} + \beta_3 \mathrm{len}_{ij} + \beta_{4k} + \varepsilon_{ijk} \qquad (S0)$$

where α_{ijk} is the overall amplification efficiency for species i at locus j for Taq k (again, expressed as the log-ratio of efficiency for species i relative to the reference species, as described in Shelton et al. 2023), which is the quantity we wish to explain. β_1, β_2, β_3, and β_{4k} are coefficients for predictor variables; and ε_{ijk} represents the error term $\varepsilon_{ijk} \sim N(0, \sigma_{ijk})$. The predictor variables are as follows: mm_{ij} is the number of mismatches summed across the forward and reverse primer binding sites for each species; GC_{ij} is the GC content of the amplicon (without the primer binding site); len_{ij} is the amplicon length (without the primer binding site) in base pairs. Note that mm_{ij}, GC_{ij}, and len_{ij} are not absolute values for each species, but rather expressed relative to the reference species *Engraulis mordax*, due to the compositional nature of metabarcoding data. For example, if species i had a total of 3 mismatches to the primer set at locus j and the reference species had 1 mismatch, mm_{ij} would be expressed as 3 – 1 = 2.

The model output of S0 showed that mismatches and length were significant predictors (95 credible interval: -0.01839, -0.01570 and 0.00034, 0.00065, respectively; Table S10), but not GC content (95 credible interval: -0.0030, 0.00032, Table S10) and so we explored the following models and determine the best fitting model:

$$\alpha_{ij} = \beta_1 \text{mm}_{ij} + \beta_2 \text{len}_{ij} + \varepsilon_{ij} \quad (S1)$$
$$\alpha_{ij} = \beta_1 \text{mm}_{ij} + \beta_2 (\text{GC}_{ij} * \text{len}_{ij}) + \varepsilon_{ij} \quad (S2)$$
$$\alpha_{ijk} = \beta_1 \text{mm}_{ij} + \beta_{2k} \text{GC}_{ij} + \beta_3 \text{len}_{ij} + \varepsilon_{ijk} \quad (S3)$$
$$\alpha_{ijk} = \beta_{1k} \text{mm}_{ij} + \beta_2 \text{len}_{ij} + \varepsilon_{ijk} \quad (S4)$$
$$\alpha_{ijk} = \beta_{2k} \text{mm}_{ij} + \alpha_2 (\text{GC}_{ij} * \text{len}_{ij}) + \varepsilon_{ijk} \quad (S5)$$
$$\alpha_{ijk} = \beta_{1k} \text{mm}_{ij} + \beta_{2k} \text{GC}_{ij} + \varepsilon_{ijk} \quad (S6)$$
$$\alpha_{ijk} = \beta_{1k} \text{mm}_{ij} + \beta_{2k} \text{GC}_{ij} + \beta_3 \text{len}_{ij} + \varepsilon_{ijk} \quad (S7)$$
$$\alpha_{ijk} = \beta_{1k} \text{mm}_{ij} + \beta_{2k} \text{GC}_{ij} + \beta_{3k} \text{len}_{ij} + \varepsilon_{ijk} \quad (S8)$$

as described above.

We ran the 'stan_lm' models with no intercept term, a Gaussian identity link function and sampling-based inference with Markov Chain Monte Carlo (MCMC), with 4000 posterior samples. The priors were set to 'NULL' which defaults to a normal distribution centered at 0 with a scale based on the data. Model summaries can be found in Table S10. For each model, we also calculated the Bayesian R^2 to assess the fit of the model. Bayesian R^2 generates posterior predictive samples, calculates the variance of predicted values and residuals for posterior samples, and averages these to give an R^2 value (which can be interpreted in the same manner as a classic R^2 where the ability of the model to explain the data ranges from 0 to 1).

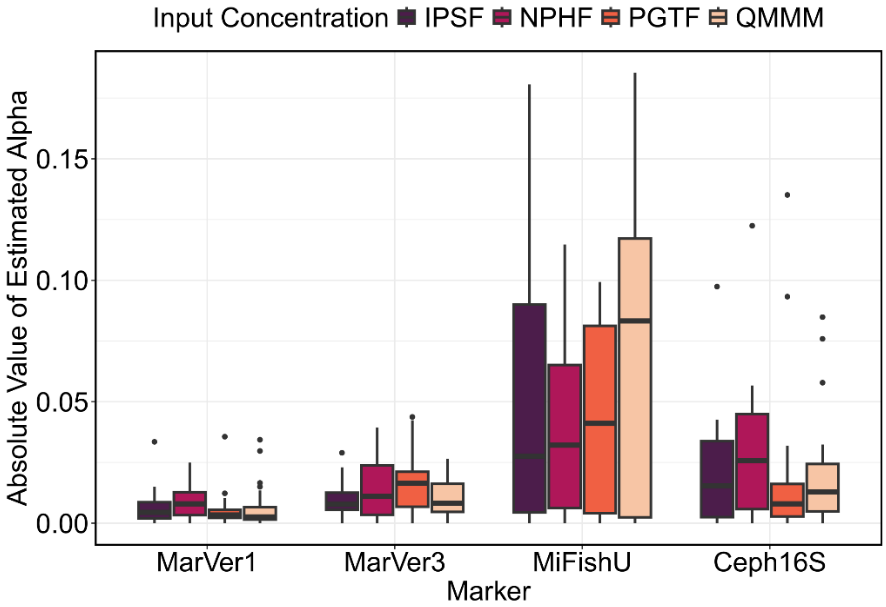

*Figure S6. Absolute value of the estimated alpha (α, amplification efficiency) from the quantitative metabarcoding model (Shelton et al., 2023) for each marker*Taq combination, using different expected proportions from mtDNA concentration for calibration for the even mock community subset.*

Table S10. Model summary for the tested models (see above) for the even mock community subset. Param are the coefficients for each model, with the mean as the average of the posterior distribution for each parameter and the SD as the standard deviation of the posterior distribution. 95% credible intervals are given with the range within which the coefficient lies with 95% probability. Rhat, or the Gelman-Rubin diagnostic, is a measure of the convergence of the MCMC chains, where a value of 1 indicates chains have converged, given with the effective number of independent samples (ESS). Bayesian R^2 also calculated for each model to assess model fit, and median R^2 reported.

Model	Param	Mean	SD	5%	95%	Rhat	ESS	R^2
S0	mm	-1.71E-02	8.16E-04	-0.01839	-0.01570	1.0008	2314	0.605
	len	4.99E-04	9.66E-05	0.00034	0.00065	1.0000	4062	
	GC	4.36E-06	1.89E-04	-0.00030	0.00032	0.9997	3946	
	TaqIPSF	-2.21E-03	2.44E-03	-0.00624	0.00164	1.0004	4027	
	TaqNPHF	-4.50E-03	2.42E-03	-0.00840	-0.00057	1.0000	4040	
	TaqPGTF	-2.25E-04	2.46E-03	-0.00424	0.00377	0.9996	3413	
S1	mm	-1.76E-02	6.90E-04	-0.01871	-0.01647	1.0033	1300	0.577
	len	5.65E-04	7.59E-05	0.00044	0.00069	0.9993	4161	
S2	mm	-1.76E-02	7.01E-04	-0.00187	-0.00165	1.0039	1312	0.579
	GC:len	-7.53E-05	1.10E-05	-0.00009	-0.00006	1.0002	4010	
S3	mm	-1.74E-02	7.69E-04	-0.01872	-0.01618	0.9994	2915	0.582
	len	5.56E-04	8.83E-05	0.00041	0.00070	0.9992	4548	
	GC:TaqIPSF	-7.59E-05	3.09E-04	-0.00060	0.00043	0.9997	3563	
	GC:TaqNPHF	1.73E-04	2.98E-04	-0.00031	0.00067	1.0006	3960	
	GC:TaqPGTF	-5.15E-04	3.01E-04	-0.00100	-0.00002	0.9997	4406	
	GC:TaqQMMM	6.08E-04	3.11E-04	0.00011	0.00112	0.9997	4303	
S4	len	5.62E-04	7.43E-05	0.00044	0.00068	1.0011	3072	0.636
	mm:TaqIPSF	-1.78E-02	1.32E-03	-0.01994	-0.01562	0.9998	3978	
	mm:TaqNPHF	-1.24E-02	1.21E-03	-0.01448	-0.01044	0.9994	4967	
	mm:TaqPGTF	-1.48E-02	1.22E-03	-0.01677	-0.01277	0.9995	3654	
	mm:TaqQMMM	-2.61E-02	1.30E-03	-0.02826	-0.02391	1.0012	2622	
S5	mm:TaqIPSF	-1.78E-02	1.31E-03	-0.02001	-0.01565	0.9995	4022	0.637
	mm:TaqNPHF	-1.25E-02	1.23E-03	-0.01451	-0.01050	1.0001	4045	
	mm:TaqPGTF	-1.47E-02	1.21E-03	-0.01672	-0.01272	0.9996	3788	
	mm:TaqQMMM	-2.61E-02	1.34E-03	-0.02840	-0.02398	1.0007	2507	
	GC:len	-7.56E-05	1.03E-05	-0.00009	-0.00006	0.9993	4975	
S6	mm:TaqIPSF	-1.71E-02	1.45E-03	-0.01944	-0.01469	0.9995	3955	0.627
	mm:TaqNPHF	-9.68E-03	1.43E-03	-0.01201	-0.00731	1.0006	4693	
	mm:TaqPGTF	-1.44E-02	1.43E-03	-0.01672	-0.01203	0.9994	4094	
	mm:TaqQMMM	-2.57E-02	1.47E-03	-0.02810	-0.02324	0.9997	2955	
	TaqIPSF:GC	4.44E-04	3.02E-04	-0.00005	0.00094	0.9999	3846	
	TaqNPHF:GC	1.41E-03	3.07E-04	0.00089	0.00191	1.0003	5708	
	TaqPGTF:GC	2.61E-04	3.11E-04	-0.00026	0.00076	0.9996	3801	
	TaqQMMM:GC	2.87E-04	3.11E-04	-0.00023	0.00079	0.9998	3739	
S7	len	5.45E-04	8.20E-05	0.00041	0.00068	0.9998	4173	0.642
	mm:TaqIPSF	-1.79E-02	1.46E-03	-0.02035	-0.01548	1.0003	3679	
	mm:TaqNPHF	-1.06E-02	1.35E-03	-0.01277	-0.00842	0.9995	5413	
	mm:TaqPGTF	-1.53E-02	1.37E-03	-0.01755	-0.01306	0.9993	4407	
	mm:TaqQMMM	-2.64E-02	1.45E-03	-0.02874	-0.02394	1.0006	3361	
	TaqIPSF:GC	-1.18E-04	3.12E-04	-0.00062	0.00039	1.0012	3658	
	TaqNPHF:GC	8.49E-04	3.00E-04	0.00035	0.00136	0.9993	5383	
	TaqPGTF:GC	-3.01E-04	3.10E-04	-0.00081	0.00021	1.0000	4295	
	TaqQMMM:GC	-1.92E-04	3.14E-04	-0.00071	0.00033	1.0000	4163	
S8	mm:TaqIPSF	-1.78E-02	1.43E-03	-0.02010	-0.01542	0.9997	4421	0.643
	mm:TaqNPHF	-1.11E-02	1.42E-03	-0.01339	-0.00881	1.0000	6618	

	mm:TaqPGTF	-1.51E-02	1.41E-03	-0.01754	-0.01284	1.0003	4302	
	mm:TaqQMMM	-2.61E-02	1.46E-03	-0.02844	-0.02373	1.0001	3684	
	TaqIPSF:GC	-3.67E-05	3.38E-04	-0.00059	0.00051	0.9995	4230	
	TaqNPHF:GC	5.61E-04	3.39E-04	0.00000	0.00111	0.9996	6960	
	TaqPGTF:GC	-2.20E-04	3.43E-04	-0.00078	0.00035	1.0003	4280	
	TaqQMMM:GC	-5.74E-05	3.39E-04	-0.00061	0.00051	1.0000	4477	
	TaqIPSF:len	4.75E-04	1.61E-04	0.00022	0.00073	0.9997	4573	
	TaqNPHF:len	8.26E-04	1.59E-04	0.00056	0.00109	0.9998	6305	
	TaqPGTF:len	4.70E-04	1.63E-04	0.00020	0.00074	1.0007	4920	
	TaqQMMM:len	4.03E-04	1.69E-04	0.00012	0.00067	0.9996	5210	

We assessed model fit using the leave-one-out cross-validation (LOO) information criterion (LOOIC), using the ‘loo’ package (Vehtari et al., 2023) in R. The approach works by systematically leaving out each data point from the dataset, refitting the model and then predicting the omitted data point; and is repeated for each datapoint. It is similar to other information criteria, where the model with the lower LOOIC values indicate a better model fit. We also assess the expected log predictive density (ELPD) and the effective number of parameters (P-LOO) to measure predictive accuracy. Higher values for ELPD indicate better model performance, and lower values of P-LOO indicate simpler models. Results from our LOO analysis are in Table S11. LOO analysis indicated that model S7 was the best fit model, which is discussed in the main paper.

Table S11. Leave-one-out cross-validation information criterion (LOO) for each model with the expected log predictive density (ELPD) and effective number of parameters (P-LOO) given. ΔELPD is the difference in ELPD from the best fitting model (using the ‘loo_compare’ function in R). Standard errors given for each measurement.

Model	LOOIC	LOOIC SE	ELPD	ELPD SE	P-LOO	P-LOO SE	ΔELPD	ΔELPD SE
S7	-1905.0	69.6	952.5	34.8	27.3	7.6	0	0
S4	-1904.7	67.7	952.4	33.8	22.4	6.4	-0.1	3.4
S8	-1903.9	69.6	951.9	34.8	29.3	7.6	-0.6	1.9
S5	-1900.0	68.3	950.4	34.2	22.5	6.2	-2.1	5.3
S6	-1870.2	60.6	935.1	30.3	22.2	5.7	-17.4	7.4
S1	-1860.7	60.5	930.4	30.2	9.4	2.4	-22.1	18.3
S3	-1860.4	60.6	930.2	30.3	13.4	2.9	-22.3	17.7
S2	-1856.9	61.0	928.4	30.5	9.7	2.3	-24.1	18.5
S0	-1698.3	72.4	849.2	36.2	15.6	3.3	-103.3	27.7

S5.2. Comparing Observation Bias Across the Even and Skewed Mock Communities

DNA metabarcoding results in compositional data, which can be difficult to interpret and make inferences from (Gloor et al., 2017). Because of this, bias imposed by PCR can be context dependent and often appear idiosyncratic among species. We therefore constructed another mock community (the skewed mock community) with the same species, but constructed using different concentrations. We amplified the skewed mock community with 12S markers, including MarVer1 (perfect matches to all species in the mock community subset) and MiFishU (some imperfect matches between primer-template in the mock community subset), using the

NEB Phusion HiFi Taq polymerase. We then compare the findings of the even community subset to the skewed community subset (Figure S7).

For MarVer1, the proportion of reads was highly correlated to the proportion of mtDNA concentration for the skewed mock community subset ($\tau = 0.90$, $p = 2.44E-15$, $n = 25$; in a similar manner to the even mock community subset, $\tau = 0.86$, $p = 9.81E-14$, $n = 26$). For the skewed community amplified by MiFishU, we observed a similar pattern to the even community, where the proportion of reads for species that contained mismatches between the primer-template were underestimated compared to expected proportions based on mtDNA concentration, but still correlated ($\tau = 0.71$, $p = 4.73E-8$, $n = 25$; compared to the even mock community subset, $\tau = 0.59$, $p = 1.09E-5$, $n = 25$).

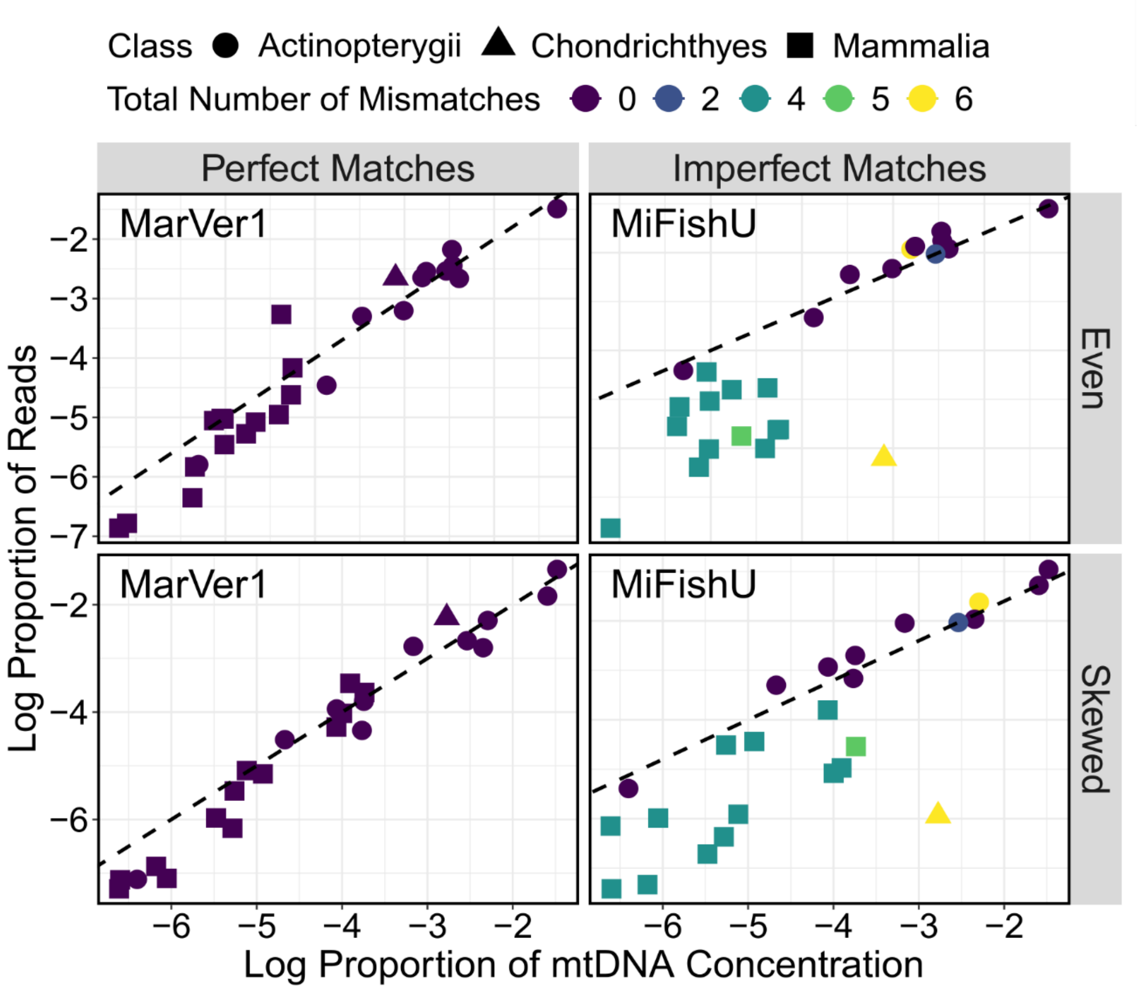

Figure S7. Log proportion of reads for the subset of species analyzed in the main paper from metabarcoding the even and skewed mock communities, with class indicated by shape and total number of mismatches (summed from the forward and reverse primer binding sites) by color.

We then determined the amplification efficiency for each species in the skewed community using the quantitative metabarcoding model (Shelton et al., 2023), as described above, for both MarVer1 and MiFishU using the expected proportions based on total genomic DNA (via

the Qubit) and mtDNA template concentration (via ddPCR). Amplification efficiency (α) was highly correlated for species between the even and skewed mock community subsets (for gDNA and mtDNA combined: $\tau = 0.78$, $p < 2.20E-16$, $n = 104$; Figure S8), as would be expected if for a given species, α were consistent across community compositions (i.e., our baseline assumption and that of Shelton et al. [2023] and McClaren et al. [2019]). There was one outlier (*Globicephala macrorhynchus*), where the even community overestimated the amplification efficiency compared to the skewed community. Interestingly, this species contained a higher proportion of reads than expected based on the mtDNA concentration in the even community, but this was not observed in the skewed community (Figure S7 – where the square Mammalia point above the 1:1 line in the perfect match, even community panel refers to *G. macrorhynchus*). We speculate this was a stochastic effect due to low template concentration of a degraded sample.

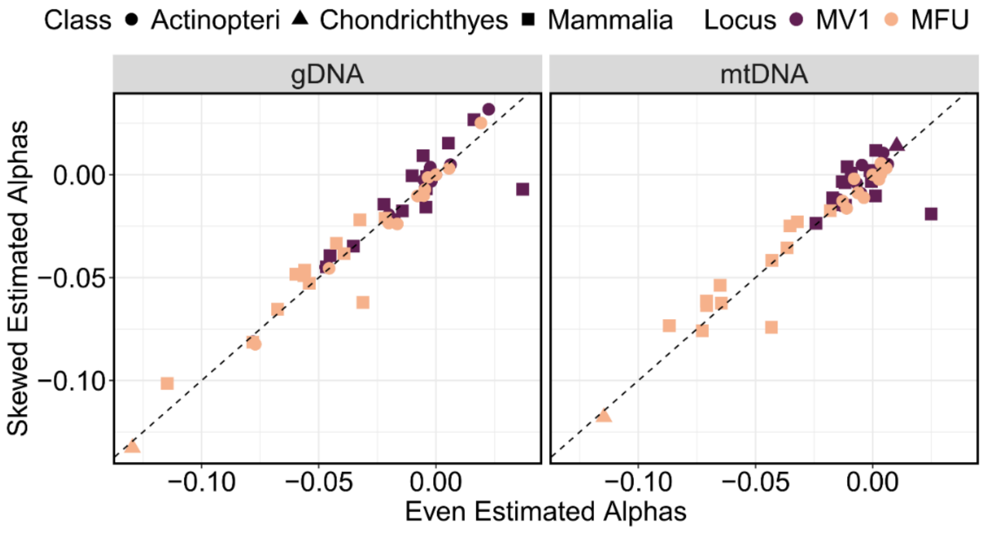

Figure S8. Amplification efficiencies (α) for each species in the even versus skewed mock community subset, by marker (color) and class (shape). Note that alphas are expressed as the log-ratio of amplification efficiencies relative to a reference species (here, Engrauils mordax).

The skewed mock community also showed the same pattern for 12S in calibration of proportions using the quantitative metabarcoding model, where mtDNA template concentration resulted in relatively lower amplification efficiency and bias compared to gDNA concentration. This was true for the primer set containing perfect matches to the primer binding sites for all species (MarVer1). For MiFishU, which contained mismatches to some species in the community, bias was driven largely by other inherent DNA characteristics for each species (Figure S9).

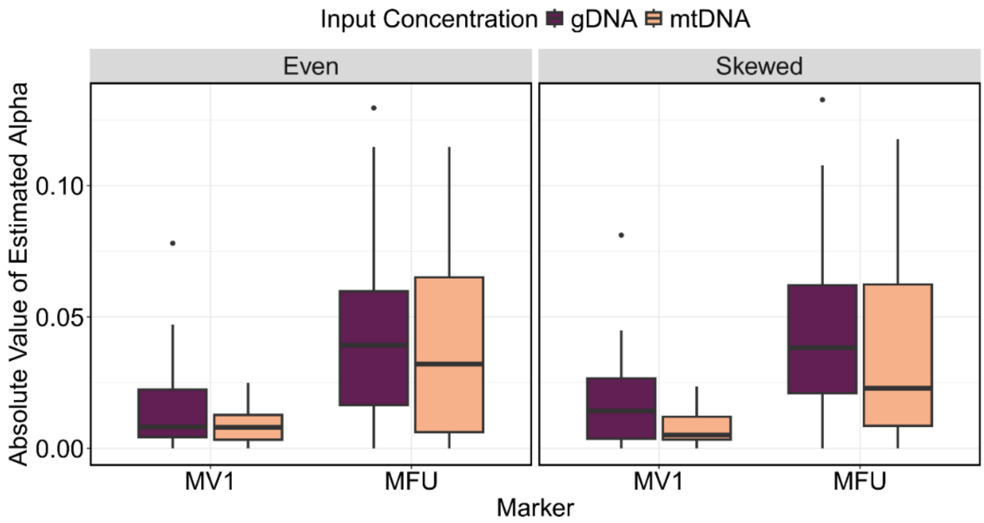

Figure S9. Absolute value of the estimated alpha (α, amplification efficiency) from the quantitative metabarcoding model (Shelton et al., 2023) for each marker, using different expected proportions for calibration (gDNA versus mtDNA), for both the even and skewed mock community subset.

S5.3. Modelling Amplification Efficiency for Species in the Skewed Mock Community Subset

We explore the predictors of amplification efficiency in a similar manner described above for the even community subset. We first calculate amplification efficiency for all species using expected proportions based on mtDNA concentrations and fit models the models below (which do not contain Taq as a predictor, as the skewed community was only run with one Taq polymerase – NEB Phusion HiFi). A preliminary model with all predictors was first run to see potential significant effects (e.g., model S9). Because GC content and mismatches were identified to be important (95% credible intervals: -0.01257, -0.00816 and .01361, 0.13878, respectively; Table S12). we fit the following models (with some the same as above). Models can be described in the same manner as above.

$$\alpha_{ij} = \beta_1 \text{mm}_{ij} + \beta_2 \text{GC}_{ij} + \beta_3 \text{len}_{ij} + \varepsilon_{ij} \quad (S9)$$

$$\alpha_{ij} = \beta_1 \text{mm}_{ij} + \beta_2 \text{len}_{ij} + \varepsilon_{ij} \quad (S1)$$

$$\alpha_{ij} = \beta_1 \text{mm}_{ij} + \beta_2 (\text{GC}_{ij} * \text{len}_{ij}) + \varepsilon_{ij} \quad (S2)$$

$$\alpha_{ij} = \beta_1 \text{mm}_{ij} + \beta_2 \text{GC}_{ij} + \varepsilon_{ij} \quad (S10)$$

$$\alpha_{ij} = \beta_1 (\text{mm}_{ij} * \text{GC}_{ij}) + \beta_2 \text{len}_{ij} + \varepsilon_{ij} \quad (S11)$$

$$\alpha_{ij} = \beta_1 (\text{mm}_{ij} * \text{GC}_{ij}) + \varepsilon_{ij} \quad (S12)$$

S5.3.1. Model Summaries and Selection

Model summaries and LOOIC analyses can be found in Tables S12 and S13. The best fitting model based off of LOOIC was model S12, which modelled amplification efficiency as a function of the interaction between mismatches and GC content. For the skewed community, the fragment length term was not important, which is consistent with the fact that the 12S

fragments did not differ in size. Furthermore, in the analysis of the even community presented in the main paper, GC content was shown to only significantly interact with the Taq NEB Phusion HiFi (95% credible interval: 0.00035, 0.00136); which is the Taq used to amplify the skewed community. This is consistent with the model output of the skewed community (95% credible interval: 0.10403, 0.13949).

Table S12. Model summary for the six tested models (see above) for the skewed mock community subset. Param are the coefficients for each model, with the mean as the average of the posterior distribution for each parameter and the SD as the standard deviation of the posterior distribution. 95% credible intervals are given with the range within which the coefficient lies with 95% probability. Rhat, or the Gelman-Rubin diagnostic, is a measure of the convergence of the MCMC chains, where a value of 1 indicates chains have converged, given with the effective number of independent samples (ESS). Bayesian R^2 also calculated for each model to assess model fit, with the median value given.

Model	Param	Mean	SD	5%	95%	Rhat	ESS	Bayes R^2
S9	mm	-1.04E-02	1.34E-03	-0.01257	-0.00816	1.0007	2542	0.645
	len	-6.87E-04	7.86E-04	-0.00197	0.00062	1.0004	1894	
	GC	7.53E-02	3.79E-02	0.01361	0.13878	1.0005	3021	
S1	mm	-1.20E-02	1.19E-03	-0.01382	-0.01001	1.0046	1588	0.636
	len	-4.20E-04	7.75E-04	-0.00168	0.00084	0.9997	1925	
S2	mm	-1.18E-02	1.15E-03	-0.01362	-0.00989	1.0002	1882	0.628
	GC:len	3.43E-04	1.04E-02	-0.01655	0.01746	1.0003	2179	
S10	mm	-1.03E-02	1.30E-03	-0.01248	-0.00824	1.0018	1887	0.621
	GC	6.88E-02	3.63E-02	0.00952	0.12851	0.9999	3127	
S11	len	-2.97E-04	7.52E-04	-0.00153	0.00095	1.0019	1630	0.660
	mm:GC	1.22E-01	1.15E-02	0.10274	0.14127	1.0058	1135	
S12	mm:GC	1.22E-01	1.10E-02	0.10403	0.13949	0.9994	2677	0.659

Table S13. Leave-one-out cross-validation information criterion (LOO) for each model for the skewed mock community, with the expected log predictive density (ELPD) and effective number of parameters (P-LOO) given. ΔELPD is the difference in ELPD from the best fitting model (using the 'loo_compare' function in R). Standard errors given for each measurement.

Model	LOOIC	LOOIC SE	elpd loo	elpd loo SE	p loo	p loo SE	elpd diff	elpd diff SE
S12	-254.7	15.7	127.3	7.9	4.4	2.1	0.0	0.0
S10	-251.1	19.1	125.5	9.6	6.8	3.9	-1.8	9.1
S11	-249.8	17.9	124.9	9.0	7.1	4.4	-2.4	1.4
S9	-248.6	18.4	124.3	9.2	8.6	4.4	-3.0	8.8
S1	-246.2	20.4	123.1	10.2	7.9	4.5	-4.2	10.3
S2	-244.4	21.5	122.2	10.7	8.8	5.0	-5.1	10.0

S6. Assessing Effects of PCR Protocols on Community Composition within the MiFishU Primer Set

In the main paper, we fit the following model using the package *zoid* (Jensen et al., 2022) in R to examine the effects of different PCR conditions (Taq polymerase, BSA and cycling profiles) on the proportion of reads:

$$Z_{ijkl} = \gamma_{0i} + \gamma_{1ij} + \gamma_{2ik} + \gamma_{3il} \quad (2)$$

where for a given PCR reaction, Z_{ijkl} is the observed proportion or read counts for species i, using Taq j, BSA treatment k, and cycling conditions l; γ_{0i} is the is the species-specific residual (intercept) term, and γ_{1ij} , γ_{2ik} and γ_{3il} are the respective terms for each predictor. We fit this model on a subset of the data that contained reads generated with MiFishU for only Acintopteri species (because the other species in the mock community were less than 0.55% of the MiFish U reads). The 95% credibility intervals for treatments that did not overlap zero (e.g., caused an increase or decrease in the proportion of reads) can be found in Table S14.

Table S14. The 95% credibility intervals (lo, hi) with mean and medians for treatment-species combination when testing the effect of Taq, BSA and touchdown PCR cycling on Actinopterygii species reads generated using MiFishU. Highlighted rows indicate species that experienced an increase or decrease in the proportion of reads (e.g., whose 95% credibility intervals did not span zero).

Species	Parameter	Mean	Median	Lo	Hi
Clupea pallasii	(Intercept)	0.993	0.992	0.830	1.152
Diogenichthys atlanticus	(Intercept)	0.377	0.375	0.192	0.563
Engralius mordax	(Intercept)	0.162	0.163	-0.040	0.357
Hippoglossus stenolepis	(Intercept)	-0.679	-0.677	-0.943	-0.440
Leuroglossus stilbius	(Intercept)	0.010	0.011	-0.187	0.201
Merluccius productus	(Intercept)	-0.364	-0.361	-0.620	-0.130
Oncorhynchus nerka	(Intercept)	-0.476	-0.474	-0.706	-0.255
Oncorhynchus tshawytscha	(Intercept)	-2.458	-2.449	-2.854	-2.081
Sardinops sagax	(Intercept)	-1.583	-1.584	-1.920	-1.263
Thaleichthys pacificus	(Intercept)	-0.459	-0.458	-0.705	-0.223
Trachurus symmetricus	(Intercept)	0.000	0.000	0.000	0.000
Clupea pallasii	**TaqIPSF**	**0.364**	**0.364**	**0.171**	**0.559**
Diogenichthys atlanticus	TaqIPSF	-0.054	-0.057	-0.283	0.180
Engralius mordax	TaqIPSF	0.030	0.031	-0.211	0.283
Hippoglossus stenolepis	TaqIPSF	-0.086	-0.087	-0.423	0.248
Leuroglossus stilbius	TaqIPSF	0.099	0.100	-0.172	0.359
Merluccius productus	**TaqIPSF**	**-0.382**	**-0.382**	**-0.675**	**-0.090**
Oncorhynchus nerka	TaqIPSF	0.148	0.152	-0.156	0.437
Oncorhynchus tshawytscha	TaqIPSF	0.359	0.361	-0.084	0.821
Sardinops sagax	TaqIPSF	0.185	0.184	-0.219	0.589
Thaleichthys pacificus	**TaqIPSF**	**-0.286**	**-0.286**	**-0.569**	**-0.006**
Trachurus symmetricus	TaqIPSF	0.000	0.000	0.000	0.000
Clupea pallasii	**TaqPGTF**	**0.329**	**0.328**	**0.142**	**0.519**
Diogenichthys atlanticus	TaqPGTF	-0.064	-0.065	-0.274	0.153
Engralius mordax	TaqPGTF	0.023	0.023	-0.213	0.259
Hippoglossus stenolepis	TaqPGTF	0.046	0.051	-0.264	0.340
Leuroglossus stilbius	TaqPGTF	0.204	0.207	-0.026	0.428
Merluccius productus	**TaqPGTF**	**-0.590**	**-0.589**	**-0.855**	**-0.318**
Oncorhynchus nerka	TaqPGTF	0.090	0.096	-0.184	0.359
Oncorhynchus tshawytscha	TaqPGTF	0.147	0.153	-0.335	0.603
Sardinops sagax	**TaqPGTF**	**0.401**	**0.401**	**0.023**	**0.777**
Thaleichthys pacificus	**TaqPGTF**	**-0.733**	**-0.732**	**-0.996**	**-0.459**
Trachurus symmetricus	TaqPGTF	0.000	0.000	0.000	0.000
Clupea pallasii	**TaqQMMM**	**0.239**	**0.238**	**0.066**	**0.412**
Diogenichthys atlanticus	TaqQMMM	-0.076	-0.075	-0.270	0.120
Engralius mordax	TaqQMMM	0.128	0.127	-0.083	0.333

Hippoglossus stenolepis	TaqQMMM	0.017	0.017	-0.255	0.297
Leuroglossus stilbius	TaqQMMM	0.201	0.203	-0.007	0.413
Merluccius productus	**TaqQMMM**	**-0.683**	**-0.684**	**-0.914**	**-0.450**
Oncorhynchus nerka	TaqQMMM	0.177	0.178	-0.073	0.416
Oncorhynchus tshawytscha	TaqQMMM	0.187	0.189	-0.231	0.603
Sardinops sagax	**TaqQMMM**	**0.422**	**0.421**	**0.094**	**0.767**
Thaleichthys pacificus	**TaqQMMM**	**-0.604**	**-0.603**	**-0.834**	**-0.384**
Trachurus symmetricus	TaqQMMM	0.000	0.000	0.000	0.000
Clupea pallasii	BSA	-0.064	-0.064	-0.227	0.097
Diogenichthys atlanticus	BSA	-0.006	-0.005	-0.201	0.188
Engralius mordax	BSA	-0.036	-0.036	-0.239	0.173
Hippoglossus stenolepis	BSA	-0.057	-0.059	-0.322	0.209
Leuroglossus stilbius	BSA	-0.074	-0.073	-0.278	0.134
Merluccius productus	**BSA**	**0.689**	**0.689**	**0.444**	**0.936**
Oncorhynchus nerka	BSA	-0.062	-0.063	-0.291	0.177
Oncorhynchus tshawytscha	BSA	-0.191	-0.195	-0.579	0.213
Sardinops sagax	BSA	-0.108	-0.106	-0.421	0.218
Thaleichthys pacificus	**BSA**	**0.944**	**0.945**	**0.698**	**1.196**
Trachurus symmetricus	BSA	0.000	0.000	0.000	0.000
Clupea pallasii	TD	0.118	0.118	-0.046	0.285
Diogenichthys atlanticus	TD	0.061	0.062	-0.139	0.265
Engralius mordax	TD	-0.036	-0.036	-0.242	0.175
Hippoglossus stenolepis	TD	-0.113	-0.116	-0.388	0.168
Leuroglossus stilbius	TD	0.052	0.050	-0.147	0.265
Merluccius productus	TD	-0.267	-0.269	-0.525	0.004
Oncorhynchus nerka	TD	-0.078	-0.076	-0.315	0.166
Oncorhynchus tshawytscha	TD	-0.135	-0.138	-0.540	0.251
Sardinops sagax	TD	-0.145	-0.143	-0.492	0.188
Thaleichthys pacificus	TD	0.127	0.127	-0.137	0.401
Trachurus symmetricus	TD	0.000	0.000	0.000	0.000

Changes in the proportion of reads for the subset mock community amplified by MiFishU, across different Taqs and cycling conditions, can be found in Figure S10. Technical replicates show reproducibility in treatment (Figure S10). For the treatments that had six replicates, we ran independent sets of three technical PCR replicates on two different MiSeq runs to determine if there were any batch and/or run effects. For the NPHF-TD treatment, the two sets of triplicates yielded slightly different proportion of reads, but note that one set of replicates had about four times the read depth than the other. When averaged out, the proportion of reads more closely match the other Taq treatments (Figure S10). For the other six replicates that contained two sets of technical PCR replicates across two runs (the QMMM-BSA treatment), read depths and proportion of reads were more similar.

The amplification efficiency for each species for each Taq-treatment combination is shown in Figure S11. The species that contained mismatches between the primer-temple (*Merluccius productus* and *Thaleichthys pacificus*) showed more variability in amplification efficiency (α) compared to those that contained perfect matches to the primer set.

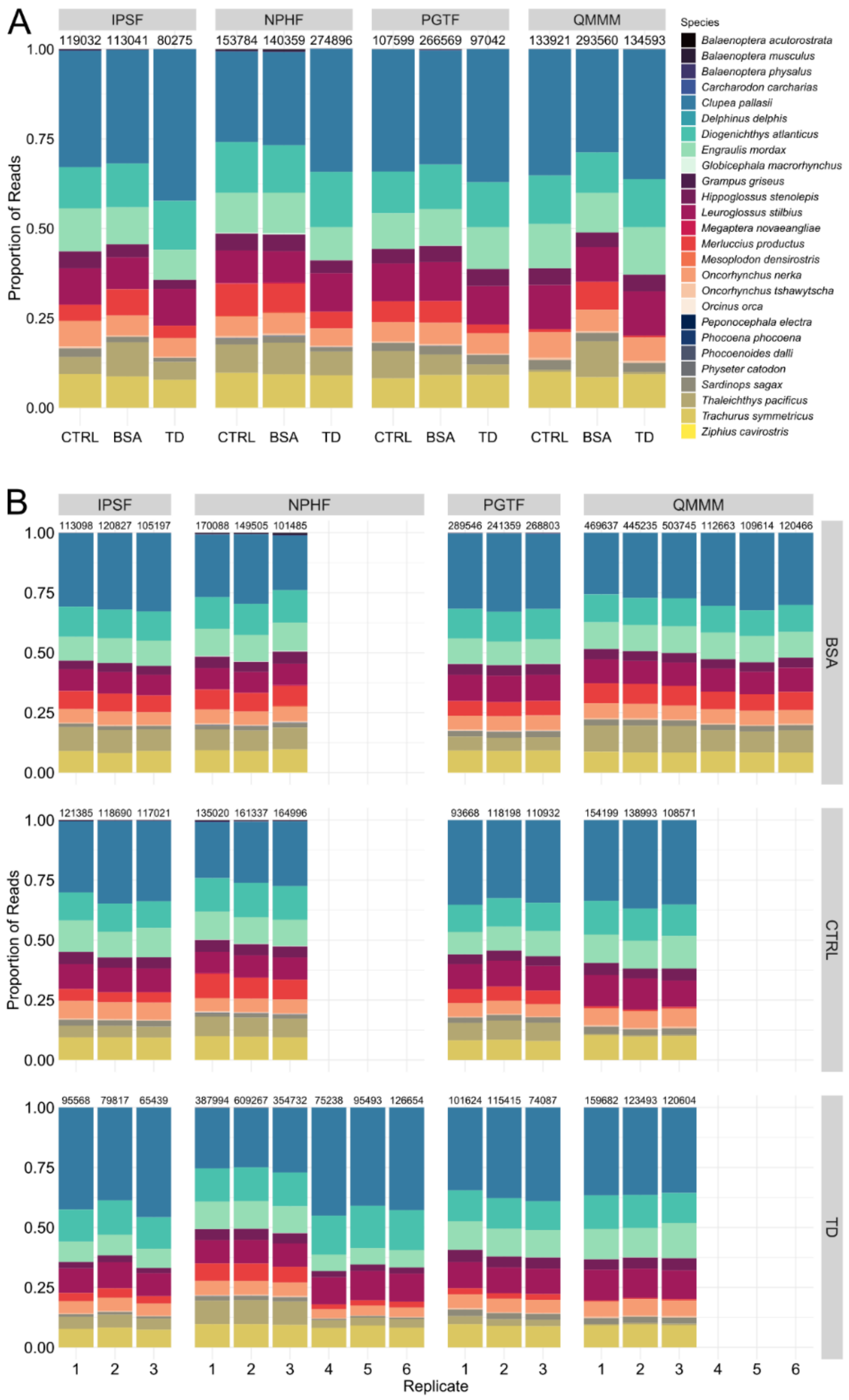

Figure S10. Proportion of reads for the even mock community subset amplified with MiFishU with different Taq polymerases and different PCR conditions. Taq polymerases are: Invitrogen Platinum Superfi (IPSF), NEB Phusion HiFi (NPHF), Promega Go Taq Flexi (PGTF) and

Qiagen Multiplex Master Mix (QMMM). PCR conditions are as follows: CTRL are the control reactions (without BSA, and normal cycling conditions); BSA are reactions with the addition of BSA and with normal cycling conditions); and TD are reactions without BSA and touchdown cycling. (A) Proportion of reads averaged across all replicates, with average read depth per sample given above bar. (B) Proportion of reads for each replicate, with read depth per sample given above bar. Note two treatments had six replicates sequenced, with each replicates 1-3 on one sequencing run and 4-6 on another run.

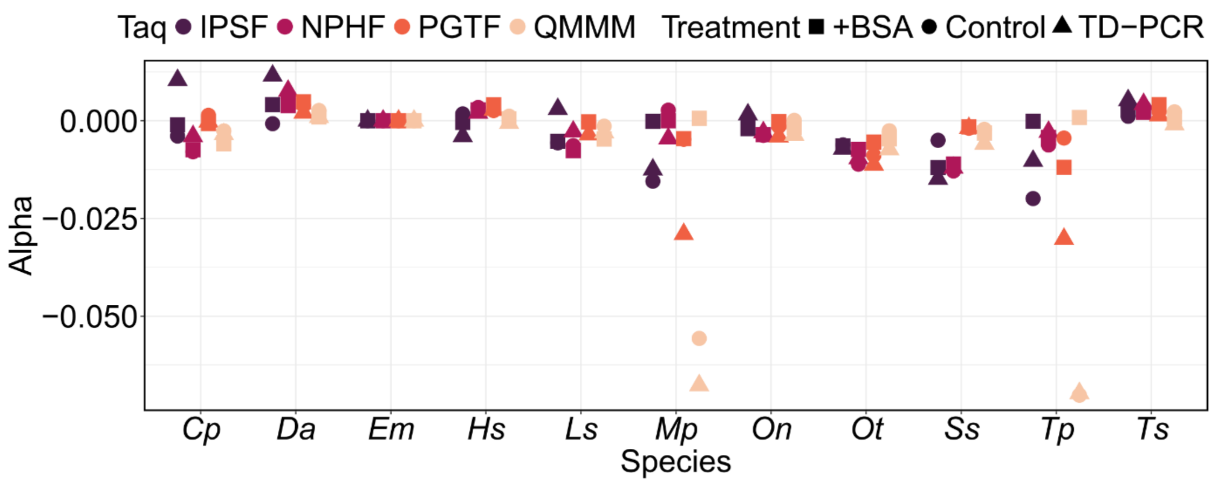

Figure S11. Amplification efficiencies (α) for the even mock community subset amplified with MiFishU with different Taq polymerases and different PCR conditions. Note that all α for each Taq-treatment combo are in relation to Em. Species are as follows: Clupea pallasii (Cp), Diogenicthys atlanticus (Da), Engraulis mordax (Em), Hippoglossus stenolepis (Hs), Leuroglossus stilbius (Ls), Merluccius productus (Mp), Oncorhynchus nerka (On), Oncorhynchus tshawytscha (Ot), Sardinops sagax (Ss), Thaleichthys pacificus (Tp), Trachurus symmetricus (Ts).

References

Altschul, S. F., Gish, W., Miller, W., Myers, E. W., & Lipman, D. J. (1990). Basic local alignment search tool. *Journal of Molecular Biology*, *215*(3), 403–410.

Archer, F. I., Morin, P. A., Hancock-Hanser, B. L., Robertson, K. M., Leslie, M. S., Bérubé, M., Panigada, S., & Taylor, B. L. (2013). Mitogenomic phylogenetics of fin whales (Balaenoptera physalus spp.): genetic evidence for revision of subspecies. *PloS One*, *8*(5), e63396.

Collins, R. A., Trauzzi, G., Maltby, K. M., Gibson, T. I., Ratcliffe, F. C., Hallam, J., Rainbird, S., Maclaine, J., Henderson, P. A., Sims, D. W., Mariani, S., & Genner, M. J. (2021). Meta-Fish-Lib: A generalised, dynamic DNA reference library pipeline for metabarcoding of fishes. *Journal of Fish Biology*, *99*(4), 1446–1454.

Dabney, J., & Meyer, M. (2011). Length and GC-biases during sequencing library amplification: A comparison of various polymerase-buffer systems with ancient and modern DNA sequencing libraries. *BioTechniques*, *52*(2), 87–94.

Gold, Z., Choi, E., Kacev, D., Frable, B., Burton, R., Goodwin, K., Thompson, A., & Barber, P. (2020). FishCARD: Fish 12S California current specific reference database for enhanced metabarcoding efforts. In *Authorea*. Authorea, Inc. https://doi.org/10.22541/au.159136805.55528691

Goodrich, B., Gabry, J., Ali, I. & Brilleman, S. (2024). rstanarm: Bayesian applied regression modeling via Stan. R package version 2.32.1 https://mc-stan.org/rstanarm.

Gloor, G.B., Macklaim, J.M., Pawlowsky-Glahn, V., & Egozcue, J.J. (2017). Microbiome datasets are compositional: and this is not optional. *Frontiers in Microbiology*, *8,* 294209.

Huber, J.A., Morrison, H.G., Huse, S.M., Neal, P.R., Sogin, M.L., Mark Welch, D.B. (2009). Effect of PCR amplicon size on assessments of clone library microbial diversity and community structure. *Environmental Microbiology*, *11*(5), 1292-1302.

Jensen, A. J., Kelly, R. P., Anderson, E. C., Satterthwaite, W. H., Shelton, A. O., & Ward, E. J. (2022). Introducing zoid: A mixture model and R package for modeling proportional data with zeros and ones in ecology. *Ecology*, 103(11), e3804.

McLaren, M. R., Willis, A. D., & Callahan, B. J. (2019). Consistent and correctable bias in metagenomic sequencing experiments. ELife, 8. https://doi.org/10.7554/eLife.46923

Nichols, R.V., Vollmers, C., Newsom, L. A., Wang, Y., Heintzman, P. D., Leighton, M., Green, R. E., & Shapiro, B. (2018) Minimizing polymerase biases in metabarcoding. Molecular Ecology Resources, 18(5), 927-939.

R Core Team (2023). R: A Language and Environment for Statistical Computing. R Foundation for Statistical Computing, Vienna, Austria. <https://www.R-project.org/>.

Rosel, P. E., Dizon, A. E., & Heyning, J. E. (1994). Genetic analysis of sympatric morphotypes of common dolphins (genus Delphinus). *Marine Biology*, 119(2), 159–167.

SantaLucia, J., Jr, & Hicks, D. (2004). The thermodynamics of DNA structural motifs. *Annual Review of Biophysics and Biomolecular Structure*, 33, 415–440.

Shelton, A. O., Gold, Z. J., Jensen, A. J., D Agnese, E., Andruszkiewicz Allan, E., Van Cise, A., Gallego, R., Ramón-Laca, A., Garber-Yonts, M., Parsons, K., & Kelly, R. P. (2023). Toward quantitative metabarcoding. *Ecology*, 104(2), e3906.

Stadhouders, R., Pas, S. D., Anber, J., Voermans, J., Mes, T. H. M., & Schutten, M. (2010). The Effect of Primer-Template Mismatches on the Detection and Quantification of Nucleic Acids Using the 5′ Nuclease Assay. *The Journal of Molecular Diagnostics: JMD*, 12(1), 109–117.

Vehtari, A., Gelman, A., Gabry, J. (2017). Practical Bayesian model evaluation using leave-one-out cross-validation and WAIC. *Statistics and Computing*, 27, 1413–1432. doi:10.1007/s11222-016-9696-4

Walsh, P. S., Metzger, D. A., & Higuchi, R. (1991). Chelex 100 as a medium for simple extraction of DNA for PCR-based typing from forensic material. *Biotechniques*, 10(4), 506-513.

Wilkinson, S. P., Davy, S. K., Bunce, M., & Stat, M. (2018). Taxonomic identification of environmental DNA with informatic sequence classification trees. In *PeerJ*. https://doi.org/10.7287/peerj.preprints.26812v1
